# Abstracts from the 20th International Symposium on Signal Transduction at the Blood-Brain Barriers

**DOI:** 10.1186/s12987-017-0071-4

**Published:** 2017-10-27

**Authors:** Andrzej Małecki, Janina Skipor-Lahuta, Michal Toborek, N. Joan Abbott, David A. Antonetti, Enming Joe Su, Daniel A. Lawrence, Müge Atış, Uğur Akcan, Canan Uğur Yılmaz, Nurcan Orhan, Poyraz Düzgün, Umut Deniz Ceylan, Nadir Arıcan, Serçin Karahüseyinoğlu, Gizem Nur Şahin, Bülent Ahıshalı, Mehmet Kaya, Sidar Aydin, Armelle Klopstein, Britta Engelhardt, Julia Baumann, Chih-Chieh Tsao, Sheng-Fu Huang, Omolara Ogunshola, Elizaveta B. Boytsova, Andrey V. Morgun, Elena D. Khilazheva, Elena A. Pozhilenkova, Yana V. Gorina, Galina P. Martynova, Alla B. Salmina, David Bueno, Jordi Garcia-Fernàndez, Victor Castro, Marta Skowronska, Michal Toborek, Matheus Uba Chupel, Luciele Guerra Minuzzi, Edith Filaire, Ana Maria Teixeira, Mariangela Corsi, Romain Versele, Andrea Fuso, Emmanuel Sevin, Cherubino Di Lorenzo, Rita Businaro, Laurence Fenart, Fabien Gosselet, Pietra Candela, Mária A. Deli, Conor Delaney, Eoin O’Keefe, Michael Farrell, Sarah Doyle, Matthew Campbell, Lester R. Drewes, A. Appelt-Menzel, A. Cubukova, M. Metzger, R. Fischer, David M. F. Francisco, Rémy Bruggmann, Alexa Fries, Kinga G. Blecharz, Josephin Wagner, Lars Winkler, Ulf Schneider, Peter Vajkoczy, Mikio Furuse, Lydia Gabbert, Christina Dilling, Dmitri Sisario, Vladimir Soukhoroukov, Malgorzata Burek, S. Guérit, E. Fidan, K. Devraj, C. J. Czupalla, J. Macas, S. Thom, K. H. Plate, H. Gerhardt, S. Liebner, András Harazin, Alexandra Bocsik, Judit Váradi, Ferenc Fenyvesi, Vilmos Tubak, Miklós Vecsernyés, Hans Christian Helms, Helle Sønderby Waagepetersen, Carsten Uhd Nielsen, Birger Brodin, Zsófia Hoyk, Melinda E. Tóth, Nikolett Lénárt, Brigitta Dukay, Ágnes Kittel, Judit Vígh, Szilvia Veszelka, Fruzsina Walter, Ágnes Zvara, László Puskás, Miklós Sántha, Sabrina Engelhardt, Omolara O. Ogunshola, Anna Huber, Alexander Reitner, Samar Osmen, Kathrin Hahn, Neli Bounzina, Anna Gerhartl, Anna Schönegger, Hannes Steinkellner, Franco Laccone, Winfried Neuhaus, Natalie Hudson, Lucia Celkova, Anne Iltzsche, Svetlana Drndarski, David J Begley, Mette Mathiesen Janiurek, Krzysztof Kucharz, Christina Christoffersen, Lars Bo Nielsen, Martin Lauritzen, Rebecca H Johnson, Dan T Kho, Simon J O’Carroll, Catherine E Angel, E. Scott Graham, Jennifer Pereira, Christina Simoglou Karali, Vinton Cheng, Niloufar Zarghami, Manuel Sarmiento Soto, Yvonne Couch, Daniel C. Anthony, Nicola R. Sibson, John Kealy, Richard F. Keep, Lisa J. Routhe, Jianming Xiang, Hong Ye, Ya Hua, Torben Moos, Guohua Xi, M. Kristensen, A. Bach, K. Strømgaard, Nikolay Kutuzov, Melissa A. Lopes-Pinheiro, Jamie Lim, Alwin Kamermans, Jack van Horssen, Wendy W.J. Unger, Ruud Fontijn, Helga E. de Vries, Petra Majerova, Ralph M. Garruto, Luca Marchetti, David Francisco, Isabelle Gruber, Ruth Lyck, Mária Mészáros, Gergő Porkoláb, Lóránd Kiss, Ana-Maria Pilbat, Zsolt Török, Zsolt Bozsó, Lívia Fülöp, Alena Michalicova, Jaroslav Galba, Sandra Mihaljevic, Michal Novak, Andrej Kovac, Yoichi Morofuji, Takashi Fujimoto, Daisuke Watanabe, Shinsuke Nakagawa, Kenta Ujifuku, Nobutaka Horie, Tsuyoshi Izumo, Takeo Anda, Takayuki Matsuo, Fang Niu, Shilpa Buch, Ádám Nyúl-Tóth, Mihály Kozma, Péter Nagyőszi, Krisztina Nagy, Csilla Fazakas, János Haskó, Kinga Molnár, Attila E. Farkas, Péter Galajda, Imola Wilhelm, István A. Krizbai, Eoin Kelly, Eugene Wallace, Chris Greene, Stephanie Hughes, John Kealy, Niamh Doyle, Marian M. Humphries, Gerald A. Grant, Alon Friedman, Ronel Veksler, Michael G. Molloy, James F. Meaney, Niall Pender, Colin P. Doherty, Minseon Park, Arkadiusz Liskiewicz, Marta Przybyla, Daniela Kasprowska-Liśkiewicz, Marta Nowacka-Chmielewska, Andrzej Malecki, Ana Pombero, Raquel Garcia-Lopez, Marta Martinez-Morga, Salvador Martinez, Ofer Prager, Lyna Solomon-Kamintsky, Karl Schoknecht, Guy Bar-Klein, Dan Milikovsky, Udi Vazana, Dror Rosenbach, Richard Kovács, Alon Friedman, Zsolt Radak, Sabela Rodríguez-Lorenzo, Remy Bruggmann, Gijs Kooij, Helga E de Vries, Semyachkina-Glushkovskaya Oxana, Bragin Denis, Vodovozova Elena, Alekseeva Anna, Salmina Alla, Salmin Vladimir, Morgun Andrey, Malinovskaya Nataliya, Khilazheva Elena, Boytsova Elizaveta, Shirokov Alexander, Navolokin Nikita, Bucharskaya Alla, Yang Yirong, Abdurashitov Arkady, Gekalyuk Artem, Ulanova Mariya, Shushunova Anastasia, Bodrova Madina, Sagatova Artem, Khorovodov Alexander, Shareef Ali Esmat, Pavlov Valery, Tuchin Artem, Kurths Jürgen, Marcelle Silva de Abreu, Ana C. Calpena, Marta Espina, Maria Luisa García, Ignacio A. Romero, David Male, Steffen Storck, Anika Hartz, Jens Pahnke, Claus U. Surma, M. Surma, Z. Giżejewski, H. Zieliński, Aleksandra Szczepkowska, Marta Kowalewska, Agata Krawczynska, Andrzej P. Herman, Janina Skipor, Nicole Kachappilly, Mike Veenstra, Rosiris Leon Rivera, Dionna W. Williams, Susan Morgello, Joan W. Berman, Ursula Wyneken, Luis Federico Batiz, Arzu Temizyürek, Rouhollah Khodadust, Mutlu Küçük, Candan Gürses, Serkan Emik, Magdalena Zielińska, Marta Obara-Michlewska, Krzysztof Milewski, Edyta Skonieczna, Inez Fręśko, Edward A. Neuwelt, Ana Raquel Santa Maria, Ana Rita Bras, Dóra Lipka, Sándor Valkai, András Kincses, András Dér, Maria A. Deli

**Affiliations:** 1 The Jerzy Kukuczka Academy of Physical Education, Katowice, Poland; 2 The Institute of Animal Physiology and Food Research, Polish Academy of Sciences, Olsztyn, Poland; 3 University of Miami, Miami, FL USA; 4 Institute of Pharmaceutical Science, King’s College London, London, United Kingdom; 5 Department of Ophthalmology and Molecular and Integrative Physiology, The University of Michigan, Ann Arbor, MI USA; 6 Department of Internal Medicine and Cardiology, The University of Michigan, Ann Arbor, MI USA; 7 School of Medicine, Department of Cellular and Molecular Medicine, Koc University, Istanbul, Turkey; 8 Department of Neuroscience, School of Medicine, Koc University, Istanbul, Turkey; 9 Department of Laboratory Animals Science, Aziz Sancar Experimental Medicine Research Institute, Istanbul University, Istanbul, Turkey; 10 Department of Neuroscience, Aziz Sancar Experimental Medicine Research Institute, Istanbul University, Istanbul, Turkey; 11 School of Medicine, Koc University, Istanbul, Turkey; 12 Department of Forensic Science, Istanbul Faculty of Medicine, Istanbul University, Istanbul, Turkey; 13 Department of Histology and Embryology, School of Medicine, Koc University, Istanbul, Turkey; 14 Department of Histology and Embryology, Istanbul Faculty of Medicine, Istanbul University, Istanbul, Turkey; 15 Department of Physiology, School of Medicine, Koc University, Istanbul, Turkey; 16 Theodor Kocher Institute, University of Bern, Bern, Switzerland; 17 Institute of Veterinary Physiology, Vetsuisse Faculty, University of Zurich, Zurich, Switzerland; 18 Zurich Center for Integrative Human Physiology, University of Zurich, Zurich, Switzerland; 19 Department of Biochemistry, Medical, Pharmaceutical and Toxicological Chemistry, Krasnoyarsk State Medical University named after Prof. V.F. Voino-Yasenetsky of the Ministry of Public Health, Krasnoyarsk, Russian Federation; 20 Research Institute of Molecular Medicine and Pathobiochemistry, Krasnoyarsk State Medical University named after Prof. V.F. Voino-Yasenetsky of the Ministry of Public Health, Krasnoyarsk, Russian Federation; 21 Department of Pediatrics, Krasnoyarsk State Medical University named after Prof. V.F. Voino-Yasenetsky of the Ministry of Public Health, Krasnoyarsk, Russian Federation; 22 Department of Pediatric Infectious Diseases, Krasnoyarsk State Medical University named after Prof. V.F. Voino-Yasenetsky of the Ministry of Public Health, Krasnoyarsk, Russian Federation; 23 Biomedical Genetics, Evolution and Development Section, Department of Genetics, Microbiology and Statistics, Faculty of Biology, University of Barcelona, Barcelona, Catalonia Spain; 24 Department of Biochemistry and Molecular Biology, University of Miami School of Medicine, Miami, FL USA; 25 Research Center for Sport and Physical Activity, Faculty of Sport Science and Physical Education, University of Coimbra, Coimbra, Portugal; 26 CIAMS, University of Orleans, Orleans, France; 27 Univ. Artois EA 2465 Laboratoire de la Barrière Hémato-Encéphalique (LBHE), Lens, France; 28 Department of Medico-Surgical Sciences and Biotechnologies, Sapienza University of Rome, Latina, Italy; 29 Department of Surgery “P. Valdoni”, Sapienza University of Rome, Rome, Italy; 30 Don Carlo Gnocchi Onlus Foundation, Milan, Italy; 31 Institute of Biophysics, Biological Research Centre, Hungarian Academy of Sciences, Szeged, Hungary; 32 Smurfit Institute of Genetics, Trinity College Dublin, Dublin, Ireland; 33 Department of Neuropathology Beaumont Hospital, Dublin 9, Ireland; 34 Department of Clinical Medicine, Trinity College Dublin, Dublin, Ireland; 35 Department of Biomedical Sciences, University of Minnesota Medical School Duluth, Duluth, MN USA; 36 Tissue Engineering and Regenerative Medicine, University Hospital Würzburg, Würzburg, Germany; 37 Fraunhofer Translational Center Würzburg ‘Regenerative Therapies in Oncology and Musculoskeletal Disease’, Würzburg, Germany; 38 Interfaculty Bioinformatics Unit and Swiss Institute of Bioinformatics, University of Bern, Bern, Switzerland; 39 Institut für Experimentelle Neurochirurgie, Charité-Universitätsmedizin Berlin, Berlin, Germany; 40 Leibnitz-Institut für Molekulare Pharmakologie im Forschungsverbund Berlin e.V, Berlin, Germany; 41 Division of Cell Structure, National Institute for Physiological Sciences, Okazaki, Japan; 42 Department of Anaesthesia and Critical Care, University of Würzburg, Würzburg, Germany; 43 Department of Biotechnology and Biophysics, University of Würzburg, Würzburg, Germany; 44 Institute of Neurology (Edinger Institute), Goethe University, Frankfurt, Germany; 45 Neurovascular Lipid Signaling Group, Pharma Center, Goethe University, Frankfurt, Germany; 46 Integrative Vascular Biology MDC & Charité Berlin, Berlin, Germany; 47 Department of Pharmaceutical Technology, Faculty of Pharmacy, University of Debrecen, Debrecen, Hungary; 48 Creative Laboratory Ltd, Szeged, Hungary; 49 Department of Pharmacy, Faculty of Health and Medical Sciences, University of Copenhagen, Copenhagen, Denmark; 50 Department of Drug Design and Pharmacology, Faculty of Health and Medical Sciences, University of Copenhagen, Copenhagen, Denmark; 51 Department of Physics, Pharmacy and Chemistry, University of Southern Denmark, Odense, Denmark; 52 Institute of Biochemistry, Biological Research Centre, Hungarian Academy of Sciences, Szeged, Hungary; 53 Institute of Genetics, Biological Research Centre, Hungarian Academy of Sciences, Szeged, Hungary; 54 Institute of Experimental Medicine, Hungarian Academy of Sciences, Budapest, Hungary; 55 Institute for Veterinary Physiology and Zurich Center for Integrative Human Physiology, University of Zurich, Zurich, Switzerland; 56 Institute of Medical Genetics, Medical University of Vienna, Vienna, Austria; 57 Department of Pharmaceutical Technology and Biopharmaceutics, University of Vienna, Vienna, Austria; 58 Competence Unit Molecular Diagnostics, AIT-Austrian Institute of Technology GmbH, Competence Center Health and Bioresources, Vienna, Austria; 59 School of Clinical Medicine, Trinity College Dublin, Dublin, Ireland; 60 National Children’s Research Centre (NCRC), Our Lady’s Children’s Hospital, Crumlin, Dublin, Ireland; 61 Center for Neuroscience, Copenhagen University, Copenhagen, Denmark; 62 Department of Clinical Biochemistry, Rigshospitalet, Copenhagen, Denmark; 63 Department of Biomedical Sciences, Copenhagen University, Copenhagen, Denmark; 64 The Faculty of Health, Aarhus University, Aarhus, Denmark; 65 Glostrup Hospital, Copenhagen, Denmark; 66 Centre for Brain Research, University of Auckland, Auckland, New Zealand; 67 Department of Pharmacology and Clinical Pharmacology, University of Auckland, Auckland, New Zealand; 68 Department of Anatomy and Medical Imaging, University of Auckland, Auckland, New Zealand; 69 School of Medical Sciences, Faculty of Medical and Health Sciences, University of Auckland, Auckland, New Zealand; 70 School of Biological Sciences, Faculty of Science, University of Auckland, Auckland, New Zealand; 71 Department of Anatomy and Medical Imaging, School of Medical Sciences, Faculty of Medical and Health Sciences, University of Auckland, Auckland, New Zealand; 72 Neurology Department, Auckland District Health Board, Auckland, New Zealand; 73 Department of Oncology, Cancer Research UK& Medical Research Council Oxford Institute for Radiation Oncology, Oxford, UK; 74 Radcliffe Department of Medicine (RDM), University of Oxford, Oxford, UK; 75 Department of Pharmacology, University of Oxford, Oxford, UK; 76 School of Biochemistry and Immunology, Trinity Biomedical Sciences Institute, Trinity College Dublin, Dublin 2, Ireland; 77 Department of Neurosurgery, University of Michigan, Ann Arbor, MI USA; 78 Department of Health Science and Technology, Aalborg University, Aalborg, Denmark; 79 Department of Neuroscience, University of Copenhagen, Copenhagen, Denmark; 80 Department of Neurophysiology, Rigshospitalet, Glostrup, Denmark; 81 Department of Pediatrics, ErasmusMC, Rotterdam, The Netherlands; 82 Department of Molecular Cell Biology and Immunology, VUmc MS Center Amsterdam, VU medical center, Amsterdam, The Netherlands; 83 Institute of Neuroimmunology, Slovak Academy of Sciences, Bratislava, Slovak Republic; 84 Graduate Program in Biomedical Anthropology, Departments of Anthropology and Biological Sciences, Binghamton University, Binghamton, NY USA; 85 Interfaculty Bioinformatics Unit, University of Bern, Bern, Switzerland; 86 Department of Medical Chemistry, University of Szeged, Szeged, Hungary; 87 AXON Neuroscience R &D Services SE, Bratislava, Slovak Republic; 88 Department of Pharmacology and Toxicology, University of Veterinary Medicine and Pharmacy, Kosice, Slovak Republic; 89 Department of Pharmaceutical Analysis and Nuclear Pharmacy, Faculty of Pharmacy of Comenius University, Bratislava, Slovak Republic; 90 Department of Neurosurgery, Nagasaki University Graduate School of Biomedical Sciences, Nagasaki, Japan; 91 Department of Medical Pharmacology, Nagasaki University Graduate School of Biomedical Sciences, Nagasaki, Japan; 92 Department of Pharmacology and Experimental Neuroscience, University of Nebraska Medical Center, Omaha, NE USA; 93 Department of Neurology, Health Care Centre Hospital 5, St James’s Hospital, Dublin 8, Ireland; 94 Department of Psychology, Beaumont Hospital, Dublin 9, Ireland; 95 Department of Neurosurgery, Stanford University School of Medicine, Stanford, California USA; 96 Department of Cognitive and Brain Sciences, Zlotowski Center for Neuroscience, Ben-Gurion University of the Negev, Beer-Sheva, Israel; 97 Department of Medical Neuroscience, Dalhousie University, Halifax, Canada; 98 Department of Medicine, University College Cork, Cork, Ireland; 99 Department of Radiology, St James’s Hospital, Dublin 8, Ireland; 100 Department of Biochemistry and Molecular Biology, Miller School of Medicine, University of Miami, Miami, FL USA; 101 Jerzy Kukuczka Academy of Physical Education, Katowice, Poland; 102 Department of Physiology, Medical University of Silesia, Katowice, Poland; 103 Department for Experimental Medicine, Medical University of Silesia, Katowice, Poland; 104 Instituto de Neurociencas de Alicante, Alicante, Spain; 105 Departments of Physiology & Cell Biology, Cognitive & Brain Sciences, the Zlotowski Center for Neuroscience, Ben-Gurion University of the Negev, Beer-Sheva, Israel; 106 Institute for Neurophysiology, Charité-University Medicine Berlin, Berlin, Germany; 107 Howard Hughes Medical Institute and Institute of Genetic Medicine, Johns Hopkins University School of Medicine, Baltimore, MD USA; 108 Department of Medical Neuroscience, Faculty of Medicine, Dalhousie University, Halifax, Canada; 109 University of Physical Education, Budapest, Hungary; 110 Department of Physiology of Human and Animals, Interdisciplinary Center of Critical Technologies in Medicine, Saratov State University, Saratov, Russian Federation; 111 Department of Pharmacy, Pharmaceutical Technology and Physicochemical, Faculty of Pharmacy and Food Sciences, University of Barcelona, Barcelona, Catalonia Spain; 112 School of Life Science, Health and Chemical Sciences, Faculty of Science, The Open University, Walton Hall, Milton Keynes, United Kingdom; 113 Institute for Pathobiochemistry, University Medical Center of the Johannes Gutenberg-University Mainz, Mainz, Germany; 114 Sanders-Brown Center on Aging Department of Pharmacology and Nutritional Sciences, University of Kentucky, Lexington, KY USA; 115 Department of Neuro-Pathology, University of Oslo (UiO) & Oslo University Hospital (OUS), Oslo, Norway; 116 Malopolska Centre of Food Monitoring, Faculty of Food Technology, University of Agriculture in Krakow, Krakow, Poland; 117 Division of Reproductive Biology, Department of Gamete and Embryo Biology, Institute of Animal Reproduction and Food Research, Polish Academy of Sciences, Olsztyn, Poland; 118 Division of Food Science, Department of Chemistry and Biodynamics of Food, Institute of Animal Reproduction and Food Research, Polish Academy of Sciences, Olsztyn, Poland; 119 Institute of Animal Reproduction and Food Research, Polish Academy of Sciences, Olsztyn, Poland; 120 The Kielanowski Institute of Animal Physiology and Nutrition, Polish Academy of Sciences, Jablonna/Warsaw, Poland; 121 Zurich Center of Integrative Human Physiology, University of Zurich, Zurich, Switzerland; 122 Institute of Veterinary Physiology, University of Zurich, Zurich, Switzerland; 123 Department of Pathology, Albert Einstein College of Medicine, Bronx, New York USA; 124 Department of Molecular and Comparative Pathobiology, Johns Hopkins University School of Medicine, Baltimore, MD USA; 125 Departments of Neurology, Neuroscience, and Pathology, Icahn School of Medicine at Mount Sinai, New York, NY USA; 126 Departments of Pathology, and Microbiology and Immunology, Albert Einstein College of Medicine, Bronx, New York USA; 127 Centro de Investigación Biomédica (CIB), Facultad de Medicina, Universidad de los Andes, Santiago, Chile; 128 Center for Life and Sciences and Technologies, Bosphorus University, Istanbul, Turkey; 129 Department of Cellular and Molecular Medicine, Medical Faculty, Koc University, Istanbul, Turkey; 130 Department of Neuroscience, Medical Faculty, Koc University, Istanbul, Turkey; 131 Department of Biotechnology, Medical Faculty, Koc University, Istanbul, Turkey; 132 Department of Neurology, Istanbul Faculty of Medicine, Istanbul University, Istanbul, Turkey; 133 Department of Chemical Technologies, Faculty of Engineering, Istanbul University, Istanbul, Turkey; 134 Neurotoxicology Department, Mossakowski Medical Research Center, Polish Academy of Sciences, Warsaw, Poland; 135 Oregon Health & Science University, Portland, OR 97239 USA; 136 Institute of Biophysics, Biological Research Centre, Hungarian Academy of Sciences, Szeged, 6726 Hungary

## A0 The 20th International Symposium on Signal Transduction at the Blood–Brain Barriers

### Andrzej Małecki^1^, Janina Skipor-Lahuta^2^, Michal Toborek ^1,3^

#### ^1^The Jerzy Kukuczka Academy of Physical Education, Katowice, Poland, ^2^The Institute of Animal Physiology and Food Research, Polish Academy of Sciences, Olsztyn, Poland, ^3^University of Miami, Miami, FL, USA

**Fig. 1:**
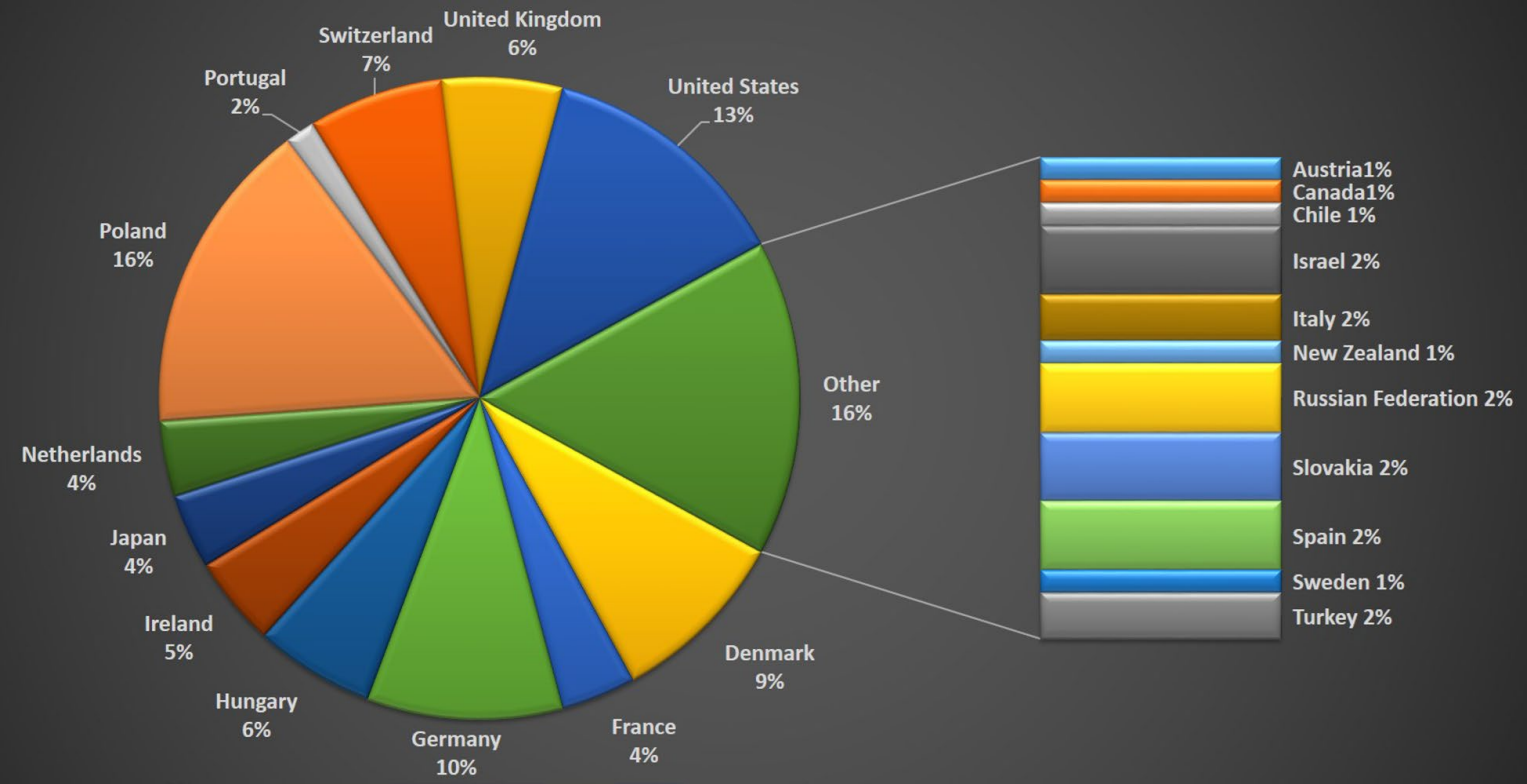
Participants of the 20th International Symposium on Signal Transduction at the Blood–Brain Barriers as divided by countries in which their research is performed

The 20th International Symposium on Signal Transduction at the Blood–Brain Barriers took place from September 13–15, 2017 in Kraków, Poland. The Organizers were the Jerzy Kukuczka Academy of Physical Education in Katowice, the Institute of Animal Reproduction and Food Research, Polish Academy of Sciences in Olsztyn as well as the Branches of the Polish Academy of Sciences in Olsztyn & Białystok and in Kraków. The Symposium Organizing Committee included Daniela Kasprowska-Liśkiewicz, Marta Nowacka-Chmielewska, Joanna Śrubarczyk, Andrzej Małecki (all from the Jerzy Kukuczka Academy of Physical Education in Katowice), Mariusz Piskuła (the President of the Olsztyn & Białystok Branch of the Polish Academy of Sciences), Janina Skipor-Lahuta, Aleksandra Szczepkowska (both from the Institute of Animal Physiology and Food Research of the Polish Academy of Sciences in Olsztyn), Irena Nalepa and Władysław Lasoń (both from Institute of Pharmacology of the Polish Academy of Sciences in Krakow). The Committee was chaired by Michal Toborek (the Jerzy Kukuczka Academy of Physical Education in Katowice and University of Miami, USA).

The Symposium program covered all areas of blood–brain barriers research with the focus on the latest developments in neurodegenerative diseases, membrane receptors and transporters, transcytosis regulators, epigenetic and transcriptional regulators, metabolic and nutrition regulation, in vivo and in vitro brain barriers models as well as the role of junctional complexes. In addition, signaling pathways implicated in the development of neurological diseases and brain tumors were addressed. The symposium aimed to encourage interactions between basic science researchers and clinicians working in academia, universities, and industry.

The program included three keynote presentations by Mikio Furuse (National Institute for Physiological Sciences, Japan) “Molecular basis of paracellular diffusion barrier”, Michal Schwartz (Weizmann Institute of Science, Israel) “Harnessing systemic immunity to combat Alzheimer’s disease”, and Britta Engelhardt (University of Bern, Switzerland) “Brain Barriers: The movers and shapers in immune privilege of the CNS”. In addition, there were 8 sessions: Session 1: Junctional complexes of the brain barriers—beyond barrier regulation, Session 2: Pathology of the brain barriers—various aspects, Session 3: Blood–brain barrier and HIV reservoirs in the CNS—formation and eradication, Session 4: Pathology of the brain barriers—experimental aspects, Session 5: Pathology of the brain barriers—clinical aspects, Session 6: Interactions between the brain barriers and stem cells in the CNS, Session 7: Brain barriers and the metabolic axis, and Session 8: Behavioral modulations of the blood–brain barrier: diet and exercise. Session 3 was sponsored by the National Institutes of Health/National Institute of Mental Health; NIH/NIMH, USA, and Session 8 was sponsored by the Institute of Animal Reproduction and Food Research, Polish Academy of Sciences, and the Jerzy Kukuczka Academy of Physical Education, Poland. In addition, there was a Poster Session.

There were 140 participants from 23 countries (Figure 1). The International Brain Barriers Society (IBBS) sponsored the Best Poster Awards. The winners were: Mette Mathiesen Janiurek (Copenhagen University, First Place), Chih-Chieh Tsao (University of Zurich, Second Place), and Alexa Fries (Charité-Universitätsmedizin Berlin, Third Place).

## A1 The puzzle of the paracellular pathway

### N. Joan Abbott

#### Institute of Pharmaceutical Science, King’s College London, London, UK

##### **Correspondence:** N. Joan Abbott (joan.abbott@kcl.ac.uk)

*Fluids and Barriers of the CNS* 2017, **14(Supp 2)**:A1

The paracellular pathway (PP) of the BBB is the extracellular cleft between adjacent endothelial cells that form the barrier. The permeability of the pathway is regulated especially by the tight junctions (TJ), supported by the adherens junctions (AJ). Many experimental studies including measuring solute flux across the BBB and transendothelial electrical resistance (TEER) indicate that the PP in brain capillaries is considerably more restricted than that of the peripheral vasculature such as skeletal and heart muscle, largely attributed to tighter TJs. Much recent work has focused on the molecular analysis of tight junction proteins, especially claudins. One main driver for this research is the need to improve drug delivery to the brain, with modulation of TJs to open up the PP a current focus.

Some topics relevant to behaviour of the PP have received less attention, such as the relation of the TJ to AJ and indeed to gap junctions between endothelial cells, the influence of the glycocalyx on the luminal surface of the endothelium and of associated cells on the basal surface (pericytes, astrocytes). Now is a good time to bring in again the ‘bigger picture’, incorporating some excellent experimental work from the past and insights from recent work, both experimental and modelling, including relating physiological function of the BBB and PP to details revealed by both molecular analysis and high resolution imaging including electron microscopy. The talk will cover cell and molecular biology in the context of the physiological functions of the BBB in helping regulate the fluid microenvironment of the brain.

## A2 Inhibiting occludin Ser490 phosphorylation prevents intracerebral hemorrhage in stroke

### David A. Antonetti ^1^, Enming Joe Su^2^, Daniel A. Lawrence^2^

#### ^1^Department of Ophthalmology and Molecular and Integrative Physiology, The University of Michigan, Ann Arbor, MI, USA, ^2^Department of Internal Medicine and Cardiology, The University of Michigan, Ann Arbor, MI, USA

##### **Correspondence:** David A. Antonetti (dantonet@med.umich.edu)

*Fluids and Barriers of the CNS* 2017, **14(Supp 2)**:A2

Stroke remains a leading cause of morbidity and mortality with limited therapeutic options. The current standard of care for patients with moderate to severe ischemic stroke is thrombolytic therapy with tissue plasminogen activator (tPA), which can significantly improve neurological outcome if given within 4 hours of stroke onset. However, later treatment with tPA increases the risk of intracerebral hemorrhage. Understanding and preventing tPA induced hemorrhage may lead to improved and extended use of thrombolytic therapy. Previous studies reveal that tPA may increase brain vascular permeability through cleavage and activation of PDGF-CC. In addition, research has demonstrated that growth factors in the VEGF and PDGF family lead to increased Ser490 phosphorylation of the tight junction protein occludin in a protein kinase C (PKC)dependent manner and mutating Ser490 to Ala (S490A) can prevent VEGF induced vascular endothelial permeability. In the current study we hypothesized that Ser490 phosphorylation was required for tPA-induced hemorrhagic transformation in the middle cerebral artery occlusion (MCAO) model of stroke. Photothrombotic MCAO was induced in C57BL/6J mice, tPA gene deletion mice or mice carrying the occludin S490A mutant at the Rosa26 locus under the CAG promoter followed by a floxed stop sequence and crossed with PDGFiCre for tamoxifen-induced vascular specific expression. MCAO induced a dramatic increase in OccS490 phosphorylation in the penumbra as determined by immunofluorescence microscopy using a P-S490 specific antibody. MCAO in tPA deleted animals, which previously were shown to prevent the increase in vascular permeability, also blocked occludin phosphorylation. Pretreatment of animals with a PKCbeta inhibitor prevented the MCAO induced permeability to dextran and prevented occludin phosphorylation. Further, expression of S490A occludin also prevented MCAO-induced vascular permeability. Importantly S490A mice also completely blocked hemorrhagic transformation in MCAO with late tPA thrombolysis (5h after MCAO). PKCbeta inhibitors also completely blocked hemorrhagic transformation induced by late tPA thrombolysis after MCAO when the kinase inhibitor was given 1h after MCAO. These results provide compelling evidence that PKCbeta phosphorylation of occludin Ser490 contributes to vascular permeability after MCAO and hemorrhagic transformation with late tPA treatment. The studies suggest that inhibition of PKCbeta may provide an opportunity to extend tPA treatment to a broader group of stroke patients.

Grant Support: This study was supported by NIH EY012021 and support from RPB to DAA and NIH HL-055374 and NS-079639 to DAL

## A3 Effects of methyl-beta-cyclodextrin on blood–brain barrier permeability in acute hypertension induced by angiotensin-II

### Müge Atış^1^, Uğur Akcan^2^, Canan Uğur Yılmaz^3^, Nurcan Orhan^4^, Poyraz Düzgün^5^, Umut Deniz Ceylan^5^, Nadir Arıcan^6^, Serçin Karahüseyinoğlu^7^, Gizem Nur Şahin^1^, Bülent Ahıshalı^8^, Mehmet Kaya^9^

#### ^1^Koc University, School of Medicine, Department of Cellular and Molecular Medicine, Istanbul, Turkey, ^2^Koc University, School of Medicine, Department of Neuroscience, Istanbul, Turkey, ^3^Istanbul University, Aziz Sancar Experimental Medicine Research Institute, Department of Laboratory Animals Science, Istanbul, Turkey, ^4^Istanbul University, Aziz Sancar Experimental Medicine Research Institute, Department of Neuroscience, Istanbul, Turkey, ^5^Koc University, School of Medicine, Istanbul, Turkey, ^6^Istanbul University, Istanbul Faculty of Medicine, Department of Forensic Science, Istanbul, Turkey, ^7^Koc University, School of Medicine, Department of Histology and Embryology, Istanbul, Turkey, ^8^Istanbul University, Istanbul Faculty of Medicine, Department of Histology and Embryology, Istanbul, Turkey, ^9^Koc University, School of Medicine, Department of Physiology, Istanbul, Turkey

##### **Correspondence:** Mehmet Kaya (mkaya942@gmail.com)

*Fluids and Barriers of the CNS* 2017, **14(Supp 2)**:A3

The loss of blood–brain barrier (BBB) integrity primarily occurs in cerebral venules and veins during acute hypertensive conditions. Methyl-beta-cyclodextrin (MBCD) causes cholesterol depletion from the cell plasma membrane and leads to caveolar transport disruption. The present study was intended to examine the effects of MBCD on the functional and structural properties of barrier type of brain microvessels in angiotensin (ANG) II–induced acute hypertension in rats. The experimental groups were designed as control, MBCD, ANG-II, and MBCD+ANG-II. BBB permeability was evaluated by determining extravasation of Evans blue (EB) and horseradish peroxidase (HRP) tracers. At five minutes after MBCD administration (5 mg/kg), acute hypertension was induced by ANG-II (60 µg/kg), and arterial blood pressure measurements were taken. ANG-II caused a significant increase in arterial blood pressure when compared with baseline values (p<0.01). The content of EB dye in the left and right cerebral cortex and left hippocampus regions of animals significantly increased in ANG-II, MBCD, and MBCD+ANG-II groups when compared with controls (p<0.05). Ultrastructurally, frequent vesicles which did not contain HRP reaction products were observed in endothelial cells of venules and veins in the cerebral cortex and hippocampus regions of brains of animals in ANG-II, and MBCD+ANG-II groups. HRP reaction products were mainly observed in astrocytes and neurons of the brain regions. Our results revealed that MBCD did not provide overall protective effects on the BBB integrity in acute hypertensive conditions and even led to BBB disruption in intact animals.

**Keywords:** Acute hypertension, Blood–brain barrier, Methyl-beta-cyclodextrin, Electron microscopy, Horseradish peroxidase, Evans blue

## A4 The role of endothelial antigen-presentation in the migration of CD8^+^Tcells across the blood–brain barrier in neuroinflammation

### Sidar Aydin, Armelle Klopstein, Britta Engelhardt

#### Theodor Kocher Institute, University of Bern, Bern, Switzerland

##### **Correspondence:** Sidar Aydin (sidar.aydin@tki.unibe.ch)

*Fluids and Barriers of the CNS* 2017, **14(Supp 2)**:A4

Multiple sclerosis (MS) is the most common inflammatory disease of the central nervous system (CNS) with unknown etiology to this date. Accumulating evidence points to a critical role of CD8^+^ T cells in MS pathogenesis. Immune cell recruitment into the CNS is controlled by the blood–brain barrier (BBB). The molecular mechanisms mediating the multi-step migration of CD8^+^ T cells across the BBB are incompletely understood. It has been suggested that endothelial antigen (Ag)-presentation contributes to CD8^+^ T-cell entry into the CNS. This prompted us to study if BBB endothelium can present Ag and if this process may contribute to CD8^+^ T-cell trafficking across the BBB. Using primary mouse brain microvascular endothelial cells (pMBMECs) as *in vitro* model for the BBB we found up-regulation of MHC-class I expression but also of the co-inhibitory molecule PD-L1 after 24 hours of stimulation with TNF-α/IFN by immunofluorescence (IF) staining. To investigate whether stimulated pMBMECs can induce Ag-dependent T-cell proliferation, we co-cultured CSFE-labeled T-cell receptor transgenic OT-I CD8^+^ T-cells recognizing the ovalbumin peptide SIINFEKL in the context of H2K^b^, with Ag-pulsed stimulated pMBMECs. Irrespective of the presence or absence of SIINFEKL, pMBMECs induced the proliferation of the naïve OT-I T cells as visualized by CFSE-dilution employing flow cytometry. Also, β2-microglobulin deficient (β2M ^-/-^) pMBMECs induced OT-I cell proliferation suggesting that pMBMECs can induce proliferation of naïve OT-I cells in an antigen and MHC class I independent fashion. At the same time we observed that OT-I cells killed WT but not β2M^-/-^pMBMECs in the presence of the SIINFEKL peptide, suggesting that full activation of OT-I effector functions needs engagement of OT-I cells with endothelial MHC class I presenting their cognate antigen. By employing in vitro live cell imaging, we finally asked if endothelial Ag-presentation contributes to the multi-step extravasation of activated OT-I cells across the BBB under physiological flow. Presence or absence of SIINFEKL peptide on MHC-class I expressing pMBMECs did not affect OT-I cell arrest on pMBMECs under physiological flow. However, in presence of SIINFEKLOT-I cell crawling was significantly reduced. This was accompanied by rapid OT-I cell mediated killing of pMBMECs under flow. Thus brain endothelial Ag-presentation triggers rapid CD8^+^ T-cell mediated killing of BBB endothelial cells under physiological flow *in vitro*. Taken together out data suggest that Ag-presentation by brain endothelial cells may lead to CD8^+^ T-cell mediated focal BBB breakdown, which is recognized as a major hallmark of MS pathogenesis.

## A5 Inhibition of Furin arrests brain endothelial cell migration and prevents TGFβ-mediated permeability changes at the blood–brain barrier

### Julia Baumann^1,2^, Chih-Chieh Tsao^1,2^, Sheng-Fu Huang^1,2^ and Omolara Ogunshola^1,2^

#### ^1^Institute of Veterinary Physiology, Vetsuisse Faculty, University of Zurich, Zurich, Switzerland, ^2^Zurich Center for Integrative Human Physiology, University of Zurich, Zurich, Switzerland

##### **Correspondence:** Julia Baumann (julia.baumann2@uzh.ch)

*Fluids and Barriers of the CNS* 2017, **14(Supp 2)**:A5

A stable well-functioning blood–brain barrier (BBB), formed by specialized endothelial cells (EC), is crucial to maintain and control cerebral homeostasis. Indeed increased barrier permeability is a common feature of numerous CNS insults and diseases. Our group has identified transforming growth factor β (TGFβ) to be involved in vessel permeability changes *in vivo.* TGFβ is known to affect cell migration but also exerts apoptotic effects giving various potential mechanisms to disrupt the barrier. TGFβ induces the expression of Furin, a ubiquitously expressed proprotein convertase that stimulates extracellular matrix degradation and cell movement. This study aimed to investigate the potential roles of TGFβ and Furin in hypoxic barrier disruption.

In a Lucifer yellow-based transwell setup exogenous TGFβ (0.25μM) increased permeability of primary endothelial cells, but had no effect on cell viability. Subsequent *in vitro* experiments were performed on rat brain microvascular ECs (RBE4) exposed to normoxia and hypoxia (1% O_2_) for 6h. EC migration tended to increase already within 6h of hypoxia and exposure to TGFβ significantly potentiated this effect. Hypoxic exposure in presence of exogenous TGFβ increased both Furin protein levels and activity. Blockade of the TGFβ-receptor I (ALK5) with 10μM SB431542 reduced Furin protein levels indicating that its activation relies on the TGFβ signaling pathway. Importantly, pharmacological inhibition of Furin with Naphthofluorescein (20μM) not only prevented hypoxic EC migration but also abrogated the TGFβ-induced potentiation. Taken together our data suggests a crucial role for Furin in mediating hypoxic and TGFβ-induced BBB permeability changes after insult. Such insights present novel potential targets for future therapy during injury and disease.

Grant Support: This work is supported by an SNF Grant 31003A_170129 to O.O

## A6 Assessment of viral inflammation in the blood–brain barrier model in vitro

### Elizaveta B. Boytsova^1,2^, Andrey V. Morgun^2,3^, Elena D. Khilazheva^1,2^, Elena A. Pozhilenkova^1,2^, Yana V. Gorina^1,2^, Galina P. Martynova^4^, Alla B. Salmina^1,2^.

#### ^1^ Dept. of Biochemistry, Medical, Pharmaceutical and Toxicological Chemistry, Krasnoyarsk State Medical University named after Prof. V.F. Voino-Yasenetsky of the Ministry of Public Health, Krasnoyarsk, Russian Federation, ^2^ Research Institute of Molecular Medicine and Pathobiochemistry, Krasnoyarsk State Medical University named after Prof. V.F. Voino-Yasenetsky of the Ministry of Public Health, Krasnoyarsk, Russian Federation, ^3^ Dept. of Pediatrics, Krasnoyarsk State Medical University named after Prof. V.F. Voino-Yasenetsky of the Ministry of Public Health, Krasnoyarsk, Russian Federation, ^4^ Dept. of Pediatric Infectious Diseases, Krasnoyarsk State Medical University named after Prof. V.F. Voino-Yasenetsky of the Ministry of Public Health, Krasnoyarsk, Russian Federation

##### **Correspondence:** Elizaveta B. Boytsova (elizaveta.boicova@mail.ru)

*Fluids and Barriers of the CNS* 2017, **14(Supp 2)**:A6

Neuroinflammation caused by infectious agents (bacteria, viruses, fungi) is an important component in the pathogenesis of numerous brain diseases. The key mechanisms of brain tissue alterations and increased permeability of the blood–brain barrier (BBB) at the loci of neuroinflammation remain insufficiently studied, thereby limiting the approaches to establish novel effective protocols for the pharmacotherapy of central nervous system inflammatory diseases. The problems of modelling neuroinflammation *in vitro* remain to be solved as well.

The aim of the study was to create an aseptic model of viral neuroinflammation *in vitro* using poly (I:C) as a viral inflammatory inducer and cerebrospinal fluid (CSF) derived from patients with viral meningitis to study some mechanisms of BBB impairment in neuroinflammation.

We used methods of molecular profiling to evaluate the expression of molecules in the BBB model *in vitro* cells involved in the response of cells to the effects of poly (I:C) and cerebrospinal fluid from patients with viral meningitis: tight gap junction proteins (ZO1, CLDN5), matrix metalloproteinase-9 (MMP9), components of inflammasome NLRP3. We applied cell biology approaches for the isolation of rat progenitor cells (neuronal precursors and astroglia), rat cerebral endothelial cells, establishment of mixed cultures (neurons, astrocytes, endothelial cells) and BBB model *in vitro*. Transendothelial electrical resistance (TEER) of endothelial cell layer was registered to evaluate BBB permeability in vitro. The study design included 6 groups. Group 1 was the BBB model *in vitro* exposed to poly (I:C). Group 2 was the BBB model *in vitro* exposed to cerebrospinal fluid obtained from patients with viral meningitis. Group 3 was a culture of cerebral endothelial cells with the addition of poly (I:C). Group 4 was a culture of endothelial cells exposed to CSF derived from patients with viral meningitis. Group 5 was a non-treated rat BBB model *in vitro*. Group 6 was a non-treated culture of rat cerebral endothelial cells.

We found that in groups 1, 2, 3, and 4, the number of cells expressing CLDN5, MMP9 and NLRP3 was higher than in the control group (p<0.05). The maximum changes have been registered in cerebral endothelial cells cultured with viral CSF: the number of cells expressing CLDN5, MMP9, NLRP3 was significantly increased comparing with group 3 (poly (I:C)) -p<0.05. However, in rat BBB model in vitro, expression of NLRP3 and MMP9 in neurons and astrocytes was higher (p<0.05) in the group with poly (I:C) than in the group 2 (CSF-treated). Interestingly, the number of endothelial cells expressing ZO1 did not differ from the control group. Nevertheless, the TEER in all the experimental groups was significantly decreased in comparison with the control group (p <0.05).

Application of the BBB model in vitro with the addition of poly (I:C) and/or cerebrospinal fluid obtained from patients with viral meningitis is useful for modelling neuroinflammation and studying the permeability of BBB.

The study is supported by the grant #16-44-243073 given by the RFBR and the Krasnoyarsk scientific foundation.

## A7 Regulation of embryonic cerebrospinal fluid composition: evolutionary implications for brain development and function

### David Bueno, Jordi Garcia-Fernàndez

#### Biomedical Genetics, Evolution and Development Section, Department of Genetics, Microbiology and Statistics, Faculty of Biology, University of Barcelona, Barcelona, Catalonia (Spain)

##### **Correspondence:** David Bueno (dbueno@ub.edu)

*Fluids and Barriers of the CNS* 2017, **14(Supp 2)**:A7

The evolution of the centralised nervous system is believed to have been a critical step in the adaptive radiation of vertebrates, especially in terms of brain structure and function. The architecture of the vertebrate brain primordium reveals the existence of connected internal cavities, the cephalic vesicles, which during foetal development become the ventricular system of the brain seen in adults. These cavities are filled with a complex, protein-rich fluid called cerebrospinal fluid (CSF). In this respect, chordates invariably possess a dorsal neural tube, while ambulacrarians exhibit an open plate-like nervous system, which is in close contact with the environment. It has been proposed that the evolutionary occurrence of a hollow nerve chord was fundamental to the origin of chordates, as the composition of the fluid in contact with the neurons no longer depended directly on the surrounding environment, but became much more strictly regulated by the activity and metabolism of the organism. Interestingly, this evolutionary process parallels developmental and regulatory aspects of some vertebrates. It has been demonstrated in higher vertebrates that embryonic CSF is key to delivering diffusible signals and nutrients to the developing brain, thereby contributing to the proliferation, differentiation and survival of neural progenitor cells, and to the expansion and patterning of the brain. Moreover, it has also been shown that the composition and homeostasis of CSF are tightly controlled from early stages of brain development, just after the closure of the anterior neuropore, before the choroid plexus (the organ producing CSF in foetuses and adults) becomes physiologically functional. Thus, this developmental process, from the closure of the neuropores to the maturation of the choroids plexus, together with the evolution of brain cavities and CSF formation constitute a fertile example of what it is called evo-devo (for evolutionary development). It has important implications for brain development and function.

## A8 Regulatory impact of occludin on the formation of HIV reservoirs in neural progenitor cells and brain pericytes

### Victor Castro, Marta Skowronska, Michal Toborek

#### Department of Biochemistry and Molecular Biology, University of Miami School of Medicine, Miami, FL, USA

##### **Correspondence:** Michal Toborek

*Fluids and Barriers of the CNS* 2017, **14(Supp 2)**:A8

HIV interactions with the cells of the blood–brain barrier (BBB) and the role of tight junction proteins in brain infection by HIV are poorly understood. HIV infection of pericytes results in biphasic changes in occludin levels, which are inversely correlated with the rate of infection. Specifically, depletion in occludin levels in the first 48 h of infection is strongly associated with an active HIV transcription and decreased expression and activation of the class-III histone deacetylase SIRT-1. Indeed, occludin has activity of a NADH oxidase that controls the expression and activation of SIRT-1. Furthermore, the viral core promoter contains two binding sites for the nuclear factor-κB (NF-κB), which plays a central role in the proviral activation pathway and stimulation of transcriptional elongation by the p65 subunit. The activity of NFκB is modulated by acetylation; thus, decreased deacylation due to lower SIRT-1 activity may drive stimulation of HIV transcription. Interestingly, decreased levels of occludin resulted in a substantial stimulation in HIV transcription rates but only limited increase in viral release, implying that far more virus is produced in pericytes than released into the medium. Similar results were obtained in neural progenitor cells, suggesting that both cell types may potentially form viral reservoirs in the brain. Indeed, at later stages of infection, elevated levels of occludin were associated with incorporation of HIV DNA into the host genome in both pericytes and neural progenitor cells. Our findings show for the first time that occludin plays an important role in HIV infection by regulating the NFκB/SIRT-1 pathway and can influence the transition between active and latent HIV infection.

This work was supported by the National Institutes of Health (NIH), grants MH072567, MH098891, HL126559, and DA039576.

## A9 Modulation of blood–brain barrier integrity through anti-inflammatory effect of combined exercise training and taurine supplementation in elderly

### Matheus Uba Chupel^1^, Luciele Guerra Minuzzi^1^, Edith Filaire^2^, Ana Maria Teixeira^1^

#### ^1^Research Center for Sport and Physical Activity, Faculty of Sport Science and Physical Education, University of Coimbra, Coimbra, Portugal, ^2^CIAMS, University of Orleans, Orleans, France

##### **Correspondence:** Matheus Uba Chupel (mtoborek@med.miami.edu)

*Fluids and Barriers of the CNS* 2017, **14(Supp 2)**:A9

Immunosenescence can lead to increased concentrations of pro-inflammatory cytokines such as IL-1β and TNF-α, which could induce blood–brain barrier (BBB) permeability in elderly people [1, 2], leading to cognitive impairment [3]. Exercise and nutritional therapies are non-pharmacological tools which emerged against immunosuppression-related diseases [4, 5]. Exercise is pointed out to modulate immune function in elderly [6, 7], however, evidence regarding long-term exercise and BBB integrity is still unknown in this population. Concerning nutrition, taurine supplementation has been investigated in humans due to its anti-inflammatory and antioxidant effects [8], nevertheless, little is known regarding its effects in BBB permeability, cognition and inflammation in elderly persons. The objective of this study was to verify the effects of exercise and supplementation with taurine on blood levels of peripheral markers of BBB permeability (S100β and neuronal specific enolase—NSE), and on IL-1β, IL-1ra, IL-17, myeloperoxidase (MPO), matrix-metalloprooteinase-9 (MMP-9) and cognition of elderly women.

48 institutionalized elderly women (83.58±6,9 years) were enrolled in the study. Subjects were divided into four groups: combined exercise training (CET: n=13), taurine supplementation (TAU: n=12), exercise with taurine (CET+TAU: n=11) and control group (CG: n=12). CET was done 2 times per week involving strength, aerobic and flexibility components. Taurine supplementation was given (1,5g/day). Interventions lasted 14 weeks, and subjects were evaluated before (T0) and after (T1) this period. Blood was collected in both moments to determine the biological markers concentration and the Mini-Mental State Examination (MMSE) was applied to examine global cognition.

There was a significant time*treatment effect on S100β, NSE and MMSE after intervention (p<0.05). S100β slightly decreased in all intervention groups, and increased in controls, where NSE levels also rose. Regression analyses including cytokines change showed that only group membership significantly determined change in S100β, while there was also a trend for change in IL-1 to be independently associated with variations in S100b (p=.054). Subsequently, univariate analyses were carried out and indicated that MPO and MMP-9 decreased in TAU group (p<0.05) and no changes were observed for the other groups. Group membership was an independent predictor of MMSE changes with variation in monocytes as an independent predictor. Meanwhile, correlation between changes showed an association between IL-17 and S100β changes.

Exercise and taurine supplementation reduced the peripheral marker of BBB permeability by an immunomodulatory mechanism, however, only the exercising elderly improved cognition.

1. Abbott NJ. Inflammatory mediators and modulation of blood–brain barrier permeability. Cell Mol Neurobiol. 2000;20: 131–47.
2. Kevin Howcroft K, Campisi J, Louis GB, Smith MT, Wise B, Wyss-Coray T, Augustine AD, McElhaney JE, Kohanski R., Sierra F. The role of inflammation in age-related disease. Aging. 2013;5: 84–93.
3. Taheri S, Gasparovic C, Huisa BN, Adair JC, Edmonds E, Prestopnik J, Grossetete M, Shah NJ, Wills J, Qualls C, Rosenberg GA. Blood–brain barrier permeability abnormalities in vascular cognitive impairment. Stroke. 2011;42: 2158–63.
4. Woods JA, Wilund KR, Martin S, Kistler BM. Exercise, inflammation and aging. Aging Dis. 2012;3: 130–40.
5. Pillon Barcelos R, Freire Royes LF, Gonzalez-Gallego J, Bresciani G. Oxidative stress and inflammation: liver responses and adaptations to acute and regular exercise. Free Rad Res. 2017;51: 222–36.
6. Simpson RJ, Lowder TW, Spielmann G, Bigley AB, LaVoy EC, Kunz H. Exercise and the aging immune system. Age Res Rev. 2012;11: 404–20.
7. Chupel MU, Direito F, Furtado GE, Minuzzi LG, Filipa M, Colado J, Ferreira JP, Filaire, Teixeira AM. Strength training decreases inflammation and increases cognition and physical fitness in older women with cognitive impairment. Front Physiol. 2017;8: 1–13.
8. Marcinkiewicz J, Kontny E. Taurine and inflammatory diseases. Amino Acids. 2014;46: 7–20.

## A10 Impact of three months ketogenic diet on brain microvessels of TgCRND8 mice

### Mariangela Corsi ^1,2^, Romain Versele ^1^, Andrea Fuso ^3^, Emmanuel Sevin^1^, Cherubino Di Lorenzo ^4^, Rita Businaro ^2^, Laurence Fenart ^1^, Fabien Gosselet ^1^, Pietra Candela ^1^

#### ^1^Univ. Artois, EA 2465, Laboratoire de la Barrière Hémato-Encéphalique (LBHE), Lens, France, ^2^Sapienza University of Rome, Dept. Of Medico-Surgical Sciences and Biotechnologies, Latina, Italy, ^3^Sapienza University of Rome, Dept. of Surgery “P. Valdoni”, Rome, Italy, ^4^ Don Carlo Gnocchi Onlus Foundation, Milan, Italy

##### **Correspondence:** Pietra Candela (pietra.candela@univ-artois.fr)

*Fluids and Barriers of the CNS* 2017, **14(Supp 2)**:A10

Alzheimer’s disease (AD) is severe progressive neurodegenerative disorder characterized by abnormal accumulation of the β-amyloid (Aβ) peptides in senile plaques as well as in the walls of intracerebral microvessels forming the blood–brain barrier (BBB). This barrier represents a tight and highly regulated barrier between the blood and the brain cells and is a key player in AD. To date, there are no effective drug therapies for AD, and the development of new alternative therapeutic approach is therefore necessary, such as nutritional strategies; one possible approach is represented by the ketogenic diet (KD), already used as a non-drug treatment for epilepsy.

KD is a diet high in fat, adequate-proteins and low carbohydrates producing ketone bodies (KBs) which represent an alternative energy source to glucose for brain cells and, unlike glucose transport, their entry across the BBB is not affected in AD. Animal studies suggest that KBs have a beneficial effect on AD by their neurotrophic effect, but also by decreasing the amount of Aβ peptides accumulation. However, why and how KBs impact brain microvessels and consequently cerebral Aβ accumulation is not well elucidated.

With this consideration in mind, it seems essential to focus on the BBB’s receptors/transporters and enzymes involved in Aβ peptide transport and metabolism, to better understand the influence of a KD on the onset and the evolution of this disease.

For 12 weeks, Wild type 129Sv (WT) and transgenic TgCRND8 (AD) mice were maintained on KD or Control Diet (CD) diets (N=6 per diet). Body weight was measured, and microvessel fractions were isolated from total brain. qPCR analyses were performed to study expression of transporters, receptors and enzymes implicated in KBs, glucose and in Aβ peptide transport and synthesis at the BBB level.

Our results showed a weight gain in AD mice after three months KD and a modulation in the expression of some Aβ transporters and enzymes at brain microvessels level. In particular in response to KD, the expression levels of these genes return to similar values to those of WT mice.

This work suggests that it may be possible to modulate Aβ transport and synthesis at the BBB level by controlling dietary intakes. However, a period of less than three months needs to be tested to evaluate the impact of this diet for a shorter period in order to be easily transfered to clinical trials.

## A11 Pericytes and BBB permeability—a co-culture model perspective

### Mária A. Deli

#### Institute of Biophysics, Biological Research Centre, Hungarian Academy of Sciences, Szeged, Hungary

##### **Correspondence:** Mária A. Deli (deli.maria@brc.mta.hu)

*Fluids and Barriers of the CNS* 2017, **14(Supp 2)**:A11

Pericytes, mural cells of small blood vessels, are highly abundant in cerebral capillaries, which form the blood–brain barrier (BBB). There is increasing evidence that pericytes are crucial for both the development and maintenance of the BBB. In many CNS pathologies, like stroke and Alzheimer’s disease pericyte dysfunction contributes to BBB damage and neuronal loss. Co-culture models are unique tools to study cellular interactions and reveal endothelial-pericyte crosstalk. We have pioneered static and dynamic blood–brain barrier co-culture models using not only two cell types, brain endothelial cells and glial cells, but also a third one, brain microvascular pericytes [1, 2]. These complex models revealed that brain pericytes are able to strengthen the barrier properties of brain endothelial cells, which is reflected by increased transendothelial resistance and decreased permeability for marker molecules. Co-culture with pericytes increases the expression and junctional localization of claudin-5, occludin and ZO-1. The expression of important BBB transporters, like BCRP (ABCG2), EAAT-1 and-2, and enzymes such as γ-glutamyl-transpeptidases, and nitric oxide synthase 1 and 2 are elevated by pericytes. Extracellular matrix components of pericytes also contribute to BBB properties and enhanced expression of tight junction proteins. The complex effects of pericytes were observed in BBB culture models from different species, including rat, mouse, porcine, monkey and human. Co-culture with pericytes increases key barrier properties, thus contributes to better BBB function of the in vitro models.

Grant support: GINOP-2.2.1-15-2016-00007; GINOP-2.3.2-15-2016-00060

1. Nakagawa S, Deli MA, Kawaguchi H, Shimizudani T, Shimono T, Kittel A, Tanaka K, Niwa M. A new blood–brain barrier model using primary rat brain endothelial cells, pericytes and astrocytes. Neurochem Int. 2009;54: 253–63
2. Walter FR, Valkai S, Kincses A, Petneházi A, Czeller T, Veszelka S, Ormos P, Deli MA, Dér A. A versatile lab-on-a-chip tool for modeling biological barriers. Sens Actuat B Chem. 2016;222:1209–19.

## A12 A role for colony stimulating factor 1 receptor signalling in the generation of cerebrovascular and BBB pathology

### Conor Delaney^1^, Eoin O’Keefe^1^, Michael Farrell^2^, Sarah Doyle^3^, Matthew Campbell^1^

#### ^1^Smurfit Institute of Genetics, Trinity College Dublin, Ireland, ^2^ Department of Neuropathology Beaumont Hospital, Ireland, ^3^Department of Clinical Medicine, Trinity College Dublin, Ireland

##### **Correspondence:** Matthew Campbell (campbem2@tcd.ie)

*Fluids and Barriers of the CNS* 2017, **14(Supp 2)**:A12

Hereditary Diffuse Leukoencephalopathy with Spheroids (HDLS) is a rare neurodegenerative leukoencephalopathy presenting clinically as cognitive dysfunction, memory deficit and motor impairment. Neuropathological examination reveals axonal swellings (spheroids), neurofilament and Ab positivity within the white matter of affected individuals. The causative gene for this monogenic dominant disorder encodes the colony stimulating factor 1 receptor (CSF1R); previously known to be involved in the differentiation, maturation and regulation of immune cells in the myeloid lineage and brain microglia, this receptor tyrosine kinase and binds two ligands CSF1 and interleukin-34 the latter of which is enriched in nervous tissue. Classically associated with macrophage function, the CSF1R gene has been revealed to have a critical role in microglial viability as well as the response of the central nervous system (CNS) to injury and stress revealing a new regulator in central nervous system (CNS) homeostasis. Recently we have identified two familial cohorts of HDLS with perivascular pathologies including amyloid beta accumulation like identical to that observed in Alzheimer’s disease, astroglial and microglial activation, and an accompanying increase in BBB permeability. Functional analyses of HDLS-variant CSF1R *in vitro* revealed conserved but reduced translation and membrane localisation, indicating that CSF1R haploinsufficiency alone may not be required for HDLS pathogenesis. We have identified aberrant post-translational processing and functionality of mutant-CSF1R and propose these altered characteristics and subsequent BBB dysfunction, rather than loss of function, may drive HDLS pathology.

## A13 Blood–brain transport pathologies: a growing challenge to the field

### Lester R. Drewes

#### Department of Biomedical Sciences, University of Minnesota Medical School Duluth, Duluth, MN, USA

##### **Correspondence:** Lester R. Drewes (ldrewes@umn.edu)

*Fluids and Barriers of the CNS* 2017, **14(Supp 2)**:A13

To maintain the brain with energy substrates and essential nutrients, endothelial cells express specific transporters of the SLC-type that are located in their luminal and/or abluminal membranes and transfer substrates down prevailing concentration gradients. (Brain endothelial cells also express ATP-dependent efflux transporters that work against concentration gradients, prevent entry of xenobiotics and drugs, and export physiological end products.) Of the more than 450 SLC-type transporters in the human genome, many are present in brain endothelial cells. Because of the critical role that endothelial cells and the neurovascular unit play in maintaining brain homeostasis, it is predicted that any disruption in transporter activity will lead to disruption of brain function and a neuropathological outcome. This, indeed, is the case and more than eight of the major blood–brain transporters, when dysfunctional in humans due to mutation, cause a serious neurological consequence ranging from microcephaly to intellectual disability. As the number of defective blood–brain transporters expands so does the challenge to find cures or treatments that ameliorate the associated conditions.

A recent addition to this list is the monocarboxylic acid transporter-1(MCT1, SLC16A1) and is documented by two reports of human subjects with MCT1 deficiency. A cohort of 96 pediatric patients with unexplained severe ketoacidosis was examined by a Dutch group [1] and subjects with both homozygous and hemizygous MCT1 inactivating mutations were identified. This study was corroborated by a report of MCT1 heterozygous Australian children who experienced similar symptoms [2]. Mutations in MCT1 may also lead to lactic acid accumulation in muscle and inhibition of contraction.

To gain new insights, MCT1 function and regulation were investigated in cells that are devoid of MCT1 or are functionally blocked by specific inhibitors. Using Seahorse technology, we have examined glycolysis and respiration in human Hap1 cells in which MCT1 is knocked out. Compared to wild type Hap1 cells, the respiratory rate for Hap1 MCT1^(**-**)^ cells is ~50% and the glycolytic rate is ~90% indicating that mitochondrial function is greatly compromised compared to glycolysis when MCT1 is not expressed. In addition, the growth (proliferation) rate of the Hap1 KO cells is about 50% of the wild type. Furthermore, no difference in mitochondrial content as determined by quantifying COX-IV expression was observed. This strongly suggests that MCT1 plays a dual role in plasma membrane transport and also mitochondria function. Not only will our results provide new insights into MCT1 function, but the findings may be transferrable to studies of other brain barrier transporters, their functions, expression regulation and the design of potential therapeutic strategies.

Grant Supported: DOD W81XWH-15-1-0060 and the UM Foundation

1. van Hasselt PM, Ferdinandusse S, Monroe GR, Ruiter JPN, Turkenburg M, et al. Monocarboxylate transporter 1 deficiency and ketone utilization. N Engl J Med. 2014;371:1900–07
2. Balasubramaniam S, Lewis B, Greed L, Meili D, Flier A, Yamamoto R, Bilić K, Till C, Sass JO. Heterozygous Monocarboxylate Transporter 1 (MCT1, *SLC16A1*) Deficiency as a Cause of Recurrent Ketoacidosis. JIMD Rep. 2016;29: 33–8.

## A14 A novel human blood–brain barrier co-culture model processed from human induced pluripotent stem cells.

### R. Fischer^1^, A. Appelt-Menzel^1, 2^, A. Cubukova^2^, M. Metzger^1, 2^

#### ^1^Fraunhofer-Institut für Silicatforschung ISC, Translationszentrum für Regenerative Therapien, Würzburg, Germany, ^2^ Fraunhofer Translational Center Würzburg ‘Regenerative Therapies in Oncology and Musculoskeletal Disease’, Würzburg, Germany

##### **Correspondence:** R. Fischer (robin.m.fischer@uni-wuerzburg.de)

*Fluids and Barriers of the CNS* 2017, **14(Supp 2)**:A14

Previously, many efforts were done to date to create a cell culture model closely mimicking the properties of the human blood–brain barrier (BBB) in vivo. By using primary cells or immortalized cell lines, only unsatisfying results comprising major deficits, could be produced so far. A promising alternative represent human induced pluripotent stem cells (hiPSC). The advantage of using hiPSC is, next to eliminating species-specific differences, the infinite and ethically inoffensive cell source that can be used as donor material. In addition, it is well-known that cell-cell communication between single players of the neurovascular unit, established through physical interaction or diffusion of certain molecules predominantly transported by extracellular vesicles, is necessary to simulate in vivo-like barrier properties. Here, we present a highly suitable, easy to culture in vitro model combining hiPSC-derived BBB endothelial cells with astrocytes, pericytes and NSCs, showing in vivo like characteristics including a transendothelial electrical resistance (TEER) up to 2,500 Ω cm^2^ and distinct upregulation of typical BBB genes i.e. tight junction proteins and BBB-specific transporters [1].

One prospective focus to obtain further knowledge about cell-cell communication and target cell modification would pose inclusion of non-destructive analysis techniques like multi electrode arrays and impedance measurements that can be implemented to optimize and simplify the model evaluation in mid and high throughput pre-clinical screening applications. Therefore, an interdisciplinary consortium of industrial and scientific research facilities recently joined within a newly BMBF-funded project named HiPSTAR (Human iPS Cell-Based BBB Technology in Alzheimer Research), combining individual expertise like organ on a chip systems, high-throughput screens of certain drug libraries, patient-specific BBB modeling or in silico prediction of the BBB. This consortium might reveal valuable new tools not only for Alzheimer research but also other related neurodegenerative and brain inflammatory disorders with increasing prevalence.

1. Appelt-Menzel A, Cubukova A, Günther K, Edenhofer F, Piontek J, Krause G, Stüber T, Walles H, Neuhaus W, Metzger M. Establishment of a human blood–brain barrier co-culture model mimicking the neurovascular unit using induced pluri- and multipotent stem cells. Stem Cell Rep. 2017;8: 894–906.

## A15 BBBHub: an expression datahub for brain barriers

### David M. F. Francisco^1^, Rémy Bruggmann^1^

#### ^1^Interfaculty Bioinformatics Unit and Swiss Institute of Bioinformatics, University of Bern, Bern, Switzerland

##### **Correspondence:** David M. F. Francisco (david.francisco@bioinformatics.unibe.ch)

*Fluids and Barriers of the CNS* 2017, **14(Supp 2)**:A15

Due to the highly specialized nature of the brain barriers tissues, as well as their development and maintenance process, transcriptome profiling is a vital tool to better understand processes in the blood–brain Barrier (BBB). In particular, RNA-seq techniques have provided the opportunity of more thorough studies in many fields of life sciences. Interestingly, the number of studies taking advantage of this technique to study the brain barriers is still very low when compared to the total number of publications concerning the brain barriers. However, we expect that (i) the number of studies using RNA-seq will increase dramatically and (ii) these methodologies will help to bring the knowledge of the BBB to a new level.

We are currently developing “BBBHub”—a web based data hub platform (i) to collect existing and new data (ii) to integrate and organize the omics-data (mainly RNA-seq) (iii) to implement data-mining tools and (iv) to provide the data to both researchers and general public. For the implementation of the BBBHub, we will use the frame work “openBIS”, an open source platform for managing scientific information, as its backbone. Furthermore, we are developing an easy-to-use front-end system to allow all researchers to use it independently of their computational expertise.

In the context of the BBBHub, we are setting up standardized data analysis workflows to allow for a more efficient comparison between datasets and are currently working on quality control determination features. These features will be very helpful to understand the utility and limitations of each dataset.

Grant support: EU H2020-MSCA-ITN-2015 675619 BtRAIN

## A16 The role of IL6 in the dysregulation of the neurovascular unit and secondary brain injury after SAH

### Alexa Fries^1^, Kinga G. Blecharz^1^, Josephin Wagner^1^, Lars Winkler^2^, Ulf Schneider^1^ and Peter Vajkoczy^1^

#### Institut für Experimentelle Neurochirurgie, Charité-Universitätsmedizin Berlin, Berlin, Germany, ^2^ Leibnitz-Institut für Molekulare Pharmakologie im Forschungsverbund Berlin e.V., Berlin, Germany

##### **Correspondence:** Alexa Fries (alexa.fries@charite.de)

*Fluids and Barriers of the CNS* 2017, **14(Supp 2)**:A16

Despite decades of research, processes leading to the intravascular inflammatory response accompanying subarachnoid hemorrhage (SAH) remain elusive. The herein presented study focusses on investigating the extent and the progression of blood–brain barrier (BBB) disruption in a SAH mouse model and the influence of proinflammatory interleukin (IL) 6 on BBB integrity and composition post SAH.

SAH was induced in wildtype (WT) and IL6 knock out (IL6KO) mice by filament perforation. BBB breakdown was evaluated by the extravasation of the fluorescent tracer Evans Blue on day 4 in SAH- and SHAM-operated mice (controls). Endothelial expression of cell contact molecules and pro-inflammatory mediators IL6, tumor-necrosis factor α (TNFα), IL1β and matrix metalloproteinase 9 (MMP9) was examined in freshly isolated cerebral capillaries employing real-time qPCR. The distribution of collagen IV as well as apoptotic pericytes (stained by desmin) were analyzed by immunofluorescence and TUNEL-Kit, respectively.

The most remarkable BBB leakage (approximately 4-fold of the control) and IL6 gene overexpression (2.7 ± 0.5-fold) was observed on day 4 due to SAH in WT mice. The pronounced expression of IL6 accompanying BBB breakdown makes this molecule interesting for further investigation. BBB leakage was significantly reduced in IL6-deficient mice. Our analysis revealed SAH-operated WT mice to show a significant decrease in the transcript level of occludin (0.5 ± 0.3-fold) but not claudin-5 (1.3 ± 0.3-fold) compared to IL6KO mice. Furthermore, we detected an upregulation of proinflammatory TNFα and IL1β in WT mice while this overexpression was attenuated in IL6KO mice (3.3 ± 1.2-fold and 2.8 ± 1.2-fold, respectively). The extracellular matrix degrading protease MMP9 was significantly elevated in SAH WT compared to IL6KO mice (2.2 ± 0.4-fold). Additionally, the MMP9 substrate collagen IV was significantly lowered in SAH WT mice (0.7 ± 0.2-fold) whereas both, SAH as well as SHAM IL6KOs showed a relatively low expression of this molecule. Finally, we noted a higher number of apoptotic pericytes in SAH-operated WT mice in comparison to IL6KOs after SAH (3.1 ± 1.0-fold).

The dynamics observed in IL6 deficient mice nominate this molecule as an important target in the reduction of BBB breakdown post SAH. Its proinflammatory influence on cerebrovascular leakage and its regulation of barrier tightening molecules implicate the critical involvement of this cytokine in second brain injury.

Grant support: This study was financially supported by Deutsche Forschungsgemeinschaft (SFB TRR43) to PV, travel cost were in part supported by Verein der Freunde und Förderer der Berliner Charité e.V.

## A17 Molecular basis of paracellular diffusion barrier

### Mikio Furuse

#### Division of Cell Structure, National Institute for Physiological Sciences, Okazaki, Japan

##### **Correspondence:** Mikio Furuse (furuse@nips.ac.jp)

*Fluids and Barriers of the CNS* 2017, **14(Supp 2)**:A17

Epithelial and endothelial tight junctions (TJs) restrict free diffusion of solutes through the paracellular pathway to separate two fluid compartments. The claudin family, which comprises more than 20 subtypes, are major structural integral membrane proteins directly involved the barrier function of TJs. A multitude of studies have revealed that part of claudin subtypes function as pores for electrolytes rather than strict barriers in TJs, and the combinations of expressed claudin subtypes determine the permeability properties of individual epithelia depending on their physiological requirements. Consistent with this concept, the loss of function not only of barrier type claudins but also of pore type claudins cause various disorders in claudin knockout mice and human hereditary diseases. In addition to TJs between adjacent cells, the paracellular pathway contains the extracellular space at tricellular contacts, where the vertices of three cells meet. These regions should also be plugged to maintain the barrier function of the cellular sheet. This task is performed by specialized structures of TJs at tricellular contacts, designated tricellular TJs (tTJs), which appear to obliterate the extracellular space there. Two types of integral membrane proteins, angulin family proteins (angulin-1/LSR, angulin-2/ILDR1 and angulin-3/ILDR2) and tricellulin are known as molecular constituents of tTJs. Angulins recruit tricellulin to tricellular contacts and both proteins are required for full barrier function of the cellular sheet. Although the impact of tTJs in epithelial functions appears less than that of TJs, angulin-2/ILDR1 knockout mice have been reported to exhibit several disorders, suggesting crucial roles of tTJ. To clarify the whole picture of the paracellular diffusion barrier, tTJs need to be studied further at a molecular level in addition to claudin-based TJs.

## A18 Protocadherins at the blood–brain barrier

### Lydia Gabbert^1^, Christina Dilling^1^, Dmitri Sisario^2^, Vladimir Soukhoroukov^2^ and Malgorzata Burek^1^

#### ^1^University of Würzburg, Department of Anaesthesia and Critical Care, Würzburg, Germany, ^2^ University of Würzburg, Department of Biotechnology and Biophysics, Würzburg, Germany

##### **Correspondence:** Malgorzata Burek (Burek_M@ukw.de)

*Fluids and Barriers of the CNS* 2017, **14(Supp 2)**:A18

Protocadherins (Pcdhs) belong to a large family of cadherin-related molecules and are organized in three large clusters, alpha, beta and gamma Pcdhs. They are highly expressed in the central nervous system (CNS). Pcdhs play a role in cell adhesion, cellular interactions and development of the CNS. Expression of Pcdhs has been well characterized in neurons, astrocytes, pericytes and in epithelial cells of the choroid plexus. Recently, we analyzed the expression of multiple Pcdhs in endothelial cells from several vascular beds. We analyzed human (hCMEC/D3) and mouse brain microvascular endothelial cell lines (BMEC) (cEND and cerebEND) as well as primary mouse BMEC. In addition, we analyzed mouse myocardial microvascular endothelial cell line (MyEND) and human umbilical vein endothelial cells (HUVEC). Using qPCR, Western blot and immunostaining we showed a strong mRNA expression of Pcdhs in all endothelial cells tested. We studied in more detail the expression of Pcdh-gamma. All members of Pcdh-gamma family can be detected with an antibody against the conserved C-terminal domain. Immunostaining of Pcdh-gamma showed diffused cytoplasmic localization in mouse BMEC. Interestingly, Pcdh-gamma protein level was higher in confluent endothelial cell cultures in comparison to non-confluent cultures. In order to study the role of Pcdh-gamma in BMEC in detail, we established a knockout of one representative Pcdh-gamma family member, PcdhgC3, using CRISPR/Cas9 system. Deletion of PcdhgC3 led to changes in the permeability and transendothelial electrical resistance (TEER). Moreover, expression of several tight junction proteins as well as transporters and cellular receptors was changed in knockout cells. The knockout cells migrated faster that the wild type cells and showed differences in adhesion to extracellular matrix components. Our results thus suggest a central role of Pcdhs in the barrier-stabilizing pathways at the blood–brain barrier. However, more studies are needed to clarify the role of Pcdhs in brain endothelial cells and other cell types of the neurovascular unit.

## A19 Astrocyte-derived Wnts regulate brain vascular permeability in mice affecting BBB maintenance

### S. Guérit ^1^, E. Fidan^1^, K. Devraj^1,2^, C.J. Czupalla^1^, J. Macas^1^, S. Thom^1^, K.H. Plate^1^, H. Gerhardt^3^, S. Liebner^1^

#### ^1^Institute of Neurology (Edinger Institute), Goethe University, Frankfurt, Germany, ^2^ Neurovascular Lipid Signaling Group, Pharma Center, Goethe University, Frankfurt, Germany, ^3^ Integrative Vascular Biology, MDC & Charité Berlin, Berlin, Germany

##### **Correspondence:** S. Liebner (stefan.liebner@kgu.de)

*Fluids and Barriers of the CNS* 2017, **14(Supp 2)**:A19

Endothelial Wnt/β-catenin signaling is necessary for developmental angiogenesis of the central nervous system (CNS) and differentiation of the blood–brain barrier (BBB), and it appears to be active at low levels also in the adult to maintain BBB characteristics. In the adult brain, pericytes and astrocytes are the closest cellular neighbours of the barrier endothelium in the neuro-vascular unit (NVU). Although both cell types participate in BBB maintenance and integrity, their contribution to Wnt growth factor release and Wnt/β-catenin signaling is poorly understood. To characterise AC-derived Wnts in BBB maintenance, we made use of GFAP-Cre:Evi^lox/lox^ mice (AC^∆Evi^), in which ACs cannot release Wnts, because the *Evi/wntless* gene is missing, which is crucial for Wnt growth factor secretion.

AC^∆Evi^ mice are viable and show no obvious gross phenotype. However, detailed analysis revealed a mild brain oedema and increased tracer permeability *in vivo*, indicating a partially dysfunctional BBB that did not affect mouse viability. Interestingly, significant oedema was observed in the cortex and subcortical regions but not in the cerebellum of AC^∆Evi^ mice, suggesting regional differences in Wnt secretion from ACs.

Analysis of vascular structure showed no significant alterations of vessel density and branching however, the structure of aquaporin-4 positive End-feet on microvessels was altered in AC^∆Evi^ mice. Electron microscopy confirmed a selective swelling of astrocyte end-feet, likely accounting for the observed BBB defect. Moreover, microvessels of AC^∆Evi^ mice showed increased vesicle incidence.

Isolated brain microvascular fragments revealed downregulated barrier-related and Wnt target genes in AC^∆Evi^ mice. Additionally, AC^∆Evi^ mice displayed structural alterations of endothelial cell-to-cell junctions.

In summary, AC^∆Evi^ mice reveal that astrocyte-derived Wnt growth factors are required to maintain the adult BBB function and cellular integrity of the NVU in an astrocyte cell-autonomous fashion.

## A20 Blood–brain barrier is protected against cytokine-induced damage by α-melanocyte stimulating hormone: a culture study

### András Harazin^1^, Alexandra Bocsik^1^, Judit Váradi^2^, Ferenc Fenyvesi^2^, Vilmos Tubak^3^, Miklós Vecsernyés^2^, Mária A. Deli^1^

#### ^1^Institute of Biophysics, Biological Research Centre, Hungarian Academy of Sciences, Szeged, Hungary, ^2^Department of Pharmaceutical Technology, Faculty of Pharmacy, University of Debrecen, Debrecen, Hungary, ^3^Creative Laboratory Ltd, Szeged, Hungary

##### **Correspondence:** András Harazin (harazin.andras@brc.mta.hu)

*Fluids and Barriers of the CNS* 2017, **14(Supp 2)**:A20

The anti-inflammatory and protective effect of the α-melanocyte stimulating hormone (αMSH) was demonstrated in animal models of diseases, such as ischemia-reperfusion or inflammations. In these pathological situations inflammatory mechanisms are triggered and maintained by proinflammatory cytokines, such as tumor necrosis factor-α (TNFα) and interleukin-1β (IL-1β) which also damage the function of biological barriers. Recently we have demonstrated, that αMSH could attenuate the inflammatory effects of TNFα and IL-1β on Caco-2 intestinal epithelial cells [1]. The effect of this neuropeptide on blood–brain barrier culture model has not been investigated yet. Our aim was to examine the effect of αMSH on cytokine-treated brain endothelial cells using a co-culture model.

Primary rat brain endothelial cells were co-cultured with rat brain pericytes and glial cells. We examined the expression of melanocortin-1 receptor (MC1R) by immunhistochemistry and RT-PCR. TNFα and IL-1β (10 ng/ml) were used to induce cell damage. Cell viability was measured by impedance (RTCA-SP, ACEA Biosciences) and MTT assay. Function of the barrier was tested by electrical resistance (TEER) and permeability measurements. Morphological changes of the junctional proteins and translocation of the inflammatory NF-κB transcription factor p65 subunit were visualized by immunhistochemistry and confocal microscopy. Reactive oxygen species production in brain endothelial cells was measured by DCFDA reagent.

We verified the presence of the MC1R mRNA and protein in brain endothelial cells. The neuropeptide αMSH (1 and 10 pM) showed no effect on rat brain endothelial cells alone, but increased the cell viability, decreased brain endothelial permeability, inhibited reactive oxygen species production, the morphological changes of junctional proteins and the nuclear translocation of NF-κB in cytokine induced damage. We demonstrated for the first time the protective effect of αMSH on cultured brain endothelial cells indicating the potential application of αMSH to protect the blood–brain barrier

Grant support: GINOP-2.2.1-15-2016-00007; GINOP-2.3.2-15-2016-00060

1. Váradi J, Harazin A, Fenyvesi F, Réti-Nagy K, Gogolák P, Vámosi G, Bácskay I, Fehér P, Ujhelyi Z, Vasvári G, Róka E, Haines D, Deli MA, Vecsernyés M. Alpha-Melanocyte Stimulating Hormone Protects against Cytokine-Induced Barrier Damage in Caco-2 Intestinal Epithelial Monolayers. PLoS ONE. 2017;12: e0170537.

## A21 Transport and metabolism of l-glutamate in brain capillary endothelial cells and astrocytes

### Hans Christian Helms^1^, Helle Sønderby Waagepetersen^2^, Carsten Uhd Nielsen^3^, Birger Brodin^1^

#### ^1^Department of Pharmacy, Faculty of Health and Medical Sciences, University of Copenhagen, Copenhagen, Denmark, ^2^ Department of Drug Design and Pharmacology, Faculty of Health and Medical Sciences, University of Copenhagen, Copenhagen, Denmark, ^3^ Department of Physics, Pharmacy and Chemistry, University of Southern Denmark, Odense, Denmark

##### **Correspondence:** Birger Brodin (Birger.brodin@sund.ku.dk)

*Fluids and Barriers of the CNS* 2017, **14(Supp 2)**:A21

The amino acid L-glutamate is an important excitatory neurotransmitter in the mammalian brain and a key player in a number of pathologies. Excessive interstitial fluid concentrations of L-glutamate cause excitotoxicity, and several mechanisms in the CNS act to keep L-glutamate levels low. The role of astrocytes in this respect is well characterized, however the role of the brain endothelial cells is still unclear. Excitatory amino acid transporters (EAAT’s of the SLC1 family) have been shown to be expressed in the abluminal membrane of brain endothelial cells from humans, rats, calves and pigs. A number of studies also indicate that the brain endothelium can mediate a vectorial transport of L-glutamate in the brain-to-blood direction. We evaluated the luminal and abluminal uptake and transendothelial transport of radiolabelled L-glutamate in electrically tight monolayers of bovine brain endothelial cells cultured either as non-contact or contact co-cultures with rat astrocytes. Both luminal and abluminal uptake followed apparent Michaelis-Menten kinetics. The abluminal uptake appeared to be dominated by EAAT-1, whereas luminal uptake was mediated via a carrier mechanism, which could not readily be identified on the molecular level. The metabolism of L-glutamate and transport of metabolites was investigated using [U-13C] L-glutamate, and it was demonstrated that intact L-glutamate, as well as metabolites were transported in the brain-to blood direction. Overall, our studies indicated that the concerted action of abluminal and luminal L-glutamate transporters mediate transendothelial transport of L-glutamate, but also that overall clearance of abluminal L-glutamate is highly dependent on metabolism.

Grant support: Birger Brodin and Hans Christian Helms would like to acknowledge the support from the Lundbeck Foundation Research Initiative on Brain Barriers and Drug Delivery (RIBBDD).

## A22 Characteristics of the blood–brain barrier in a novel model of vascular neurodegeneration

### Zsófia Hoyk^1#^, Melinda E. Tóth^2#^, Nikolett Lénárt^2^, Brigitta Dukay^2^, Ágnes Kittel^4^, Judit Vígh^1^, Szilvia Veszelka^1^, Fruzsina Walter^1^, Ágnes Zvara^3,^ László Puskás^3^, Mária A. Deli^1#^, Miklós Sántha^1#^

#### ^1^Institute of Biophysics, Biological Research Centre, Hungarian Academy of Sciences, Szeged, Hungary, ^2^Institute of Biochemistry, Biological Research Centre, Hungarian Academy of Sciences, Szeged, Hungary, ^3^Institute of Genetics, Biological Research Centre, Hungarian Academy of Sciences, Szeged, Hungary, ^4^Institute of Experimental Medicine, Hungarian Academy of Sciences, Budapest, Hungary

##### **Correspondence:** Zsófia Hoyk (zsofi@brc.hu) and Mária A. Deli (deli.maria@brc.mta.hu)

^#^Zsófia Hoyk, Melinda E. Tóth, Mária A. Deli and Miklós Sántha contributed equally to this work

*Fluids and Barriers of the CNS* 2017, **14(Supp 2)**:A22

High triglyceride levels are known risk factors for cardio- and cerebrovascular diseases. A growing number of evidence suggests that dyslipidemia is associated with neurodegenerative diseases, too. However, data are lacking on the cellular and molecular characteristics of the neurovascular unit in hypertriglyceridemia. In the present study neurovascular changes—brain vessel permeability, gene expression changes and morphology of the cells of the neurovascular unit in the hippocampus and frontal cortex—were examined in ApoB-100 transgenic mice, a model of human atherosclerosis. Increased extravasation of the small molecular marker fluorescein was observed in the hippocampus of transgenic mice, but not in the frontal cortex. No change was seen for albumin permeability between the groups or brain regions. The most striking change in gene expression was a drop in the homeobox gene Meox2 and the docosahexaenoic acid transporter Mfsd2a in the transgenic animals. By immunohistochemistry no difference was observed in the staining pattern of the tight junction proteins claudin-5 and occludin in brain microvessels and of microglia cells stained for marker Iba-1comparing the transgenic mice with their wild-type littermates. Astroglia cells in the frontal cortex, in contrast, showed marked alterations. The GFAP immunoreactivity showed two types of staining pattern: it was detected in astroglial cell bodies and processes and in structures closely associated with brain capillaries but not linked to glial cells in wild-type animals, while in ApoB-100 transgenic mice the predominant GFAP immunostaining revealed a cellular pattern. Quantitative analysis of the area fractions and average size of GFAP and Iba-1 immunoreactive structures revealed no difference between wild-type and transgenic mice. Vimentin immunoreactivity was typically observed along brain capillaries in the frontal cortex of wild-type animals. Electron microscopicy analysis of brain capillaries showed an increase in the ratio of discontinuous tight junctions and edematous astrocytes in both the frontal cortex and hippocampus in ApoB-100 transgenic mice compared with wild-type animals. We conclude that the increased blood–brain barrier permeability, changes in genes regulating brain vessel integrity and tightness, and morphological changes in astroglia cells closely associated with brain capillaries might be linked in ApoB-100 transgenic mice.

Grant support: OTKA-NN111006, GINOP-2.3.2-15-2016-00060, GINOP-2.3.2-15-2016-00001

## A23 A new insight into the blood–brain barrier: glutathione crosstalk between astrocytes and endothelial cells

### Sheng-Fu Huang, Sabrina Engelhardt, Omolara O. Ogunshola

#### Institute for Veterinary Physiology and Zurich Center for Integrative Human Physiology, University of Zurich, Zurich, Switzerland

##### **Correspondence:** Sheng-Fu Huang (sheng-fu.huang@uzh.ch)

*Fluids and Barriers of the CNS* 2017, **14(Supp 2)**:A23

Glutathione (GSH) is a thiol compound present in high concentrations in cells of all organs’ it has many physiological functions including drug detoxification and defense against cell stress. Notably, in aging human brain and many neurological disorders and diseases it has been shown that GSH is below normal levels suggesting GSH is essential to support brain function. In the brain, astrocytes (ACs) are the major GSH producers and the cellular link between neurons and the blood–brain barrier (BBB). ACs secrete GSH to support neuronal survival but the contributions of GSH to BBB integrity are still unknown. The BBB is formed by endothelial cells (EC) that strictly control exchange between blood and brain compartment using tight junctions, maintaining brain homeostasis. Our recent metabolomics data showed that GSH levels are higher in AC than EC during hypoxia/ischemia stimulating us to investigate GSH crosstalk between AC and EC under stress. Our results show that exogenous GSH improves EC tight junction localization and maintains EC morphology during 48h of normoxia and hypoxia/ischemia. Furthermore, exogenous GSH prevented hypoxia/ischemia-induced EC barrier impairment suggesting it benefits barrier stability. Interestingly, ACs rapidly secrete GSH during 16h of hypoxia and ischemia. In correlation, mRNA levels of the GSH transporters (MRP2 and MRP4) significantly increase in AC between 6h and 24h of hypoxia/ischemia. Moreover, the γ-glutamyl transferase mRNA, an extracellular GSH stabilizer, was upregulated after 6h hypoxia/ischemia. These evidences indicate that ACs increase the secretion and stability of GSH during hypoxia/ischemia. We are currently tracking isotope labeled endogenous GSH movement in our AC-EC co-culture system to confirm GSH shuttling from AC to endothelial cells. Such knowledge will provide potential candidates to develop novel strategies to reduce BBB impairment and brain injury.

Grant support: Swiss National Science Foundation grant 31003A_170129 to O.O

## A24 The blood–brain barrier is altered during Rett Syndrome: Implications for Rett-therapeutics

### Anna Huber^1,2^, Alexander Reitner^1,2^, Samar Osmen^1,3^, Kathrin Hahn^3^, Neli Bounzina^1^, Anna Gerhartl^3^, Anna Schönegger^1^, Hannes Steinkellner^1^, Franco Laccone^1^, Winfried Neuhaus^2,3^

#### ^1^Institute of Medical Genetics, Medical University of Vienna, Vienna, Austria., ^2^Department of Pharmaceutical Technology and Biopharmaceutics, University of Vienna, Vienna, Austria., ^3^AIT-Austrian Institute of Technology GmbH, Competence Center Health and Bioresources, Competence Unit Molecular Diagnostics, Vienna, Austria.

##### **Correspondence:** Winfried Neuhaus (winfried.neuhaus@ait.ac.at)

*Fluids and Barriers of the CNS* 2017, **14(Supp 2)**:A24

The Rett syndrome (RTT) is a pervasive developmental disorder, primarily affecting girls with a prevalence of 1 in every 10,000 live births. It represents the second most common cause of intellectual disability in females. Children with RTT develop normally until 6–12 months of life. However, they subsequently lose learned skills like language and purposeful hand use, and develop irregular respirations in the wake period, gait abnormalities and seizures. RTT is known to be caused in 95% of the cases by sporadic de novo loss-of-function mutations in the *MECP2* gene. In recent years the focus of research expanded from neurons (which express MeCP2 to the highest content) to other CNS cells such as astrocytes and confirmed their mechanistic relevance for the disease. Despite the importance of the blood–brain barrier (BBB) for drug delivery of Rett-therapeutics and although it is known that the BBB functionality is changed during several neurodegenerative diseases, no data exist about the BBB and possible functional alterations during RTT until now. Therefore, we studied the presence and functional relevance of MeCP2 at the BBB by comparing wild-type to MeCP2 knock-out mice. According to our results, MeCP2 is present in mouse brain endothelial cells shown in brain sections, isolated capillaries and isolated cells as well as in human cells of the neurovascular unit. After evaluation of tight junction and transporter molecule expression *in vivo*, significant differences could be found indicating changes of BBB functionality during RTT, which could be confirmed *in vitro*. Recombinant TAT-MeCP2 is currently under development as a therapeutic protein. Comprehensive uptake and functional in vitro as well as *in vivo* evaluation studies about the potential of TAT-MeCP2 as a drug were accomplished. In addition, an *in vitro* BBB model was established to evaluate the BBB permeability of different TAT-MeCP2 preparations in order to lead and optimize the production process.

In summary, our data indicate that the BBB is changed during RTT and this probably influences CNS accessibility for drugs. Moreover, RTT is a novel example for a neurodegenerative disease with altered BBB functionality. However, it has still to be investigated if this altered BBB functionality is causally linked to the disease progression.

Grant support: This work was supported by the Germans parents association for children with RETT syndrome (“Elternhilfe für Kinder mit RETT-Syndrom”).

## A25 Circadian regulation of the inner retinal vasculature; A paradigm for geographic atrophy development

### Natalie Hudson^1^, Lucia Celkova^1^, Sarah Doyle^2,3^, Matthew Campbell^1^

#### ^1^Smurfit Institute of Genetics, Trinity College Dublin, Dublin, Ireland, ^2^School of Clinical Medicine, Trinity College Dublin, Dublin, Ireland, ^3^National Children’s Research Centre (NCRC), Our Lady’s Children’s Hospital, Crumlin, Dublin, Ireland

##### **Correspondence:** Matthew Campbell (campbem2@tcd.ie)

*Fluids and Barriers of the CNS* 2017, **14(Supp 2)**:A25

Circadian rhythms are intrinsic biological processes that occur in 24 hour oscillations. These enable an organism to anticipate and prepare for regular environmental changes and are regulated by external cues such as light and temperature. Photoreceptor outer segment (POS) phagocytosis is one process that occurs daily upon light onset. The involvement of circadian rhythms in retinal function, however, is still not fully elucidated. Here, we wished to study the role of circadian clock components in the regulation of processes directly related to the replenishment of shed POS’.

Wild-type C57BL6/J mice (10-12 weeks) were sacrificed at 8AM, 2PM, 8PM and 2AM (8AM and 8PM corresponding to 12 h lights on: 12 h lights off cycle) for *in vivo* analyses. Retinal protein and mRNA was extracted and tight junction (TJ) and circadian clock components analysed by western blotting and qRT-PCR, respectively. For verification that the response was circadian rather than diurnal, further cohorts of C57BL6/J mice were housed in either 24 h of darkness or dark-adapted for three weeks, in which the light cycle was reversed, and subsequently sacrificed and retinal protein and mRNA analysed as above. Serum shock experiments were found to re-establish circadian rhythms in *in vitro* primary human retinal microvascular endothelial cells.

We found that TJ component claudin-5 cycled in the retinal vasculature throughout the day and the clock component BMAL1 was required for claudin-5 cycling. Claudin-5 transcript and protein levels cycled throughout the day in a circadian-dependent, rather than diurnal manner, with lower levels at 8PM compared to 8AM. These changes phenotypically led to more permeable retinal vessels in the evening compared to morning as observed by fundus fluorescein angiography (FFA) and dynamic contrast enhanced magnetic resonance imaging (DCE-MRI) analyses. The observed effects were similarly seen in other wild-type mouse strains. Circadian-regulated changes in retinal vascular permeability was not evident in BMAL1^FL/FL^-Tie-2 mice, where the clock gene BMAL1 was lacking in endothelial cells. Mice exposed to a high fat diet in tandem with persistent suppression of claudin-5 developed rapid onset of a geographic atrophy (GA) like phenotype, which is the end stage of the condition dry age-related macular degeneration (AMD).

We have discovered that the inner blood-retinal barrier is highly dynamic and plays a critical role in replenishing POS. Circadian regulation of claudin-5 facilitates the exchange of material between blood and the neural retina. Therefore, regulating claudin-5 or circadian clock components may represent a novel therapeutic target for treating GA.

This work has been supported by: Science Foundation Ireland and Health Research Board

## A26 Novel insights into the blood–brain barrier crossing ability of Apo-E and polysorbate-80 modified human serum albumin nanoparticles and their distribution within the CNS: a possible strategy for protein and other therapeutic delivery to the brain

### Anne Iltzsche, Svetlana Drndarski, David J. Begley

#### Institute of Pharmaceutical Science, Kings College London, London, United Kingdom

##### **Correspondence:** David J. Begley (david.begley@kcl.ac.uk)

*Fluids and Barriers of the CNS* 2017, **14(Supp 2)**:A26

Human serum albumin (HSA) nanoparticles, modified with apolipoprotein E (ApoE) or a polysorbate-80 coating, cross the blood–brain barrier (BBB) *in vivo* after intra-jugular injection. The biodegradable and non-antigenic nanoparticles were produced at a size of 200nm diameter using the desolvation method and their surface modified with ApoE or polysorbate-80.

Electron microscopy showed the brain intracellular localization of the modified nanoparticles 15 min after intra-jugular injection. Whereas unmodified HSA nanoparticles were not seen within the brain tissue. The BBB crossing ability of the modified nanoparticles is mediated by receptor-mediated endocytosis followed by transcystosis involving the LRP1 receptor as demonstrated *in vitro*. However, the rapid speed of nanoparticle movement within the brain tissue remains surprising. Confocal microscopy confirmed electron microscopic findings, underlining the rapid movement of the modified HSA nanoparticles through the brain tissue following brain BBB endothelial cell internalization. Furthermore, it allowed for the precise quantification of the number of nanoparticles per cell in the brain and calculation of injected dose subsequently found in the brain. Surface modified HSA nanoparticles were located over 4μm from the closest vessel 15 min after injection. With an average size of 200 nm the nanoparticles are far too big to travel freely through the highly tortuous extracellular space. Furthermore, the modified nanoparticles were never seen extracellularly within brain, except for the basal lamina. Astrocytic end-feet covering the brain endothelial cells are well positioned to mediate further transport through the brain tissue. Cytoplasmic flow rates of 2-16 mm/hr would explain the rapid rate of intracellular movement observed. Furthermore, the modified HSA nanoparticles were highly concentrated in the astrocytic end-feet 5 min after injection, shown by confocal microscopy, and localized in the distal astrocytic processes 15 min after injection, as shown by 3D reconstruction. Within 30 minutes the HSA nanoparticles are distributed within the cytoplasm of neurones. These observations lead to a re-evaluation of the role of astrocytes in the rapid movement of large structures within the brain.

Quantification of the polysorbate-80 coating revealed a monomolecular surfactant coverage of the nanoparticles. Investigating the protein corona developed on the nanoparticle surface upon contact with serum, with and without polysorbate-80 coating, is crucial in elucidating the biological identity and handling of the nanoparticles *in vivo*. SDS-PAGE and LC-MS analysis of the protein corona revealed a highly complex corona composition of adsorbed serum proteins including the presence of multiple LDL(LRP)-receptor ligands. An in depth understanding of the intracellular transport mechanism utilized by ApoE and polysorbate-80 modified HSA nanoparticles bears great promise for the future design of CNS targeted drug delivery systems for large protein-based therapeutics.

## A27 The role of the sphingosine-1-phosphate and its signalling pathway in modulation of the blood–brain barrier integrity in mice

### Mette Mathiesen Janiurek^1^, Krzysztof Kucharz^1^, Christina Christoffersen^2,3^, Lars Bo Nielsen^4^, and Martin Lauritzen^1,5^

#### ^1^Center for Neuroscience, Copenhagen University, Copenhagen, Denmark, ^2^Department of Clinical Biochemistry, Rigshospitalet, Copenhagen, Denmark, ^3^Department of Biomedical Sciences, Copenhagen University, Copenhagen, Denmark, ^4^The Faculty of Health, Aarhus University, Aarhus, Denmark, ^5^Glostrup Hospital, Copenhagen, Denmark

##### **Correspondence:** Martin Lauritzen (martin.johannes.lauritzen@regionh.dk)

*Fluids and Barriers of the CNS* 2017, **14(Supp 2)**:A27

Modulation of endogenous signalling pathways that regulate the blood–brain barrier (BBB) permeability represents a potential strategy for increased drug delivery to the brain. Sphingosine-1-phosphate receptors (S1PRs) are surface membrane receptors located on both the lumenal and ablumenal side of endothelial cells. In a healthy organism, the majority of the S1PR agonist, i.e. sphingosine-1-phosphate (S1P), is transported in the blood by the molecular chaperone, apolipoprotein-M (ApoM). Disruption of the ApoM-S1P signalling, i.e. by lack of ApoM expression, is associated with an impaired endothelial barrier in the lungs, but its significance for the BBB integrity is not fully understood.

Aim: To determine the importance of the S1P/S1PR signaling pathway for endothelial function and BBB permeability.

We used in vivo two-photon fluorescence microscopy in ApoM^-/-^ mice with impaired ApoM/S1P signalling and wild type littermates. To monitor BBB permeability in a living brain, water-soluble fluorophores of varying sizes, i.e. 0.365kDa Sodium Fluorescein, 0.643kDa Alexa488 and 10KDa FITC-dextran were injected intravenously. The accumulation of the fluorophores was measured in real time in the brain parenchyma at a depths ranging from 0 to 120 μm relative to the brain surface.

Loss of ApoM-mediated S1P transport leads to increased permeability towards blood-circulating fluorescent molecules. Compared to wild-type littermates, the BBB in ApoM^-/-^ mice exhibited a 5-fold increase in the accumulation of small fluorophores (<0.65kDa, i.e. Sodium Fluorescein and Alexa488) within 30 minutes post injection. This effect was not observed with larger molecules, i.e. 10kDa FITC-dextran. Moreover, pharmacological modulation of S1P/S1PR pathway with S1PR agonist SEW2871 leads to reversal of the phenotype and partially reinstated the BBB integrity.

The BBB integrity towards blood circulating molecules can be altered by a targeted modulation of the S1P/S1PR/ApoM signalling, implying that this pathway plays an important role in maintaining the integrity of the BBB. Alteration of the BBB by disrupting ApoM/S1P/S1PR signaling may be a novel target for bidirectional modulation of the BBB integrity for increased trans-BBB drug delivery to CNS. These findings are of relevance for understanding the mechanisms of neurodegeneration in chronic diseases, as well as for rescuing the BBB function in acute brain disorders.

## A28 The functional and inflammatory response of brain endothelial cells to toll-like receptor agonists

### Rebecca H Johnson^1,2^, Dan T Kho^1,2^, Simon J O’Carroll^1,3^, Catherine E Angel^4^, E. Scott Graham^1,2^.

#### ^1^Centre for Brain Research, University of Auckland, Auckland, New Zealand, ^2^ Department of Pharmacology and Clinical Pharmacology, University of Auckland, Auckland, New Zealand, ^3^ Department of Anatomy and Medical Imaging, University of Auckland, Auckland, New Zealand, School of Medical Sciences, Faculty of Medical and Health Sciences, University of Auckland, Auckland, New Zealand, ^4^ School of Biological Sciences, Faculty of Science, University of Auckland, Auckland, New Zealand

##### **Correspondence:** E. Scott Graham (s.graham@auckland.ac.nz)

*Fluids and Barriers of the CNS* 2017, **14(Supp 2)**:A28

Toll-Like Receptors (TLRs) represent an important early warning mechanism for the immune system to detect infection or tissue damage, however there has been limited research investigating how different TLR ligands function in human brain microvascular endothelial cells. The focus of this research was to determine the downstream neuro-inflammatory responses of various TLR ligands and their effect on endothelial barrier strength. A range of commercially available TLR ligands were tested for their pharmacological response profiles using xCELLigence Biosensor technology which revealed that the brain endothelial cells responded to the TLR3 ligand Poly(I:C), the TLR 4 ligand LPS and the FDA approved TLR7 ligand Imiquimod, but not to any other TLR agonists. Using Cytometric Bead Array it was found that whilst Poly(I:C) and LPS induced a pronounced pro-inflammatory response, Imiquimod did not induce the secretion of any pro-inflammatory cytokines. ECIS technology was used to determine temporal changes in endothelial barrier strength, which revealed that both Poly(I:C) and LPS have a similar effect on barrier integrity as TNFα. In contrast, Imiquimod was able to induce a rapid and sustained increase in barrier strength. In addition to this, it was further observed that Imiquimod was able to abrogate the production of some but not all pro-inflammatory cytokines and chemokines secreted during inflammatory activation of the brain endothelial cells. PCR analysis revealed that the brain endothelial cells lacked TLR7, and further xCELLigence experiments revealed that the novel response to Imiquimod was not replicated with a panel of alternative TLR7 ligands. This finding indicates that novel receptors exist for Imiquimod (polypharmacology), which do not mediate a pro-inflammatory signal but can attenuate various pro-inflammatory responses including TNα, TLR3 and TLR4 agonists. The challenge now is to identify these immunosuppressive receptors and assess their therapeutic potential.

## A29 Real-time biosensor technology reveals the temporal dynamic changes in BBB endothelial barrier strength following exposure to inflammatory cytokines present in the serum of MS patients

### Rebecca H. Johnson^1,2^, Sarah Gough^1^, Dan T. Kho^1,2^, Simon J. O’Carroll^1,3^, Catherine E. Angel^4^, Jennifer Pereira^5^, E. Scott Graham^1,2^

#### ^1^Centre for Brain Research, University of Auckland, Auckland, New Zealand, ^2^ Department of Pharmacology and Clinical Pharmacology, University of Auckland, Auckland, New Zealand, ^3^ Department of Anatomy and Medical Imaging, School of Medical Sciences, Faculty of Medical and Health Sciences, University of Auckland, Auckland, New Zealand, ^4^ School of Biological Sciences, Faculty of Science, University of Auckland, Auckland, New Zealand, ^5^ Neurology Department, Auckland District Health Board, Auckland, New Zealand

##### **Correspondence:** E. Scott Graham (s.graham@auckland.ac.nz)

*Fluids and Barriers of the CNS* 2017, **14(Supp 2)**:A29

In this research we have investigated the temporal regulation of brain endothelial barrier function following exposure to a variety of inflammatory mediators elevated in the serum of MS patients. It is well known that Relapsing Remitting MS is a chronic inflammatory disease with evidence of autoimmune dysfunction, therefore our serum cytokine panel was biased towards Th1, Th17 and leukocyte recruitment/activation mediators. In the MS sera, we detected elevated levels of (i) IL-1β, IL2, IL-4, IL6, IL-12, IL17, IFNs, (ii) the chemokines fractalkine, MCP-1, IP-10, RANTES, IP-10, MIP1α, and (iii) various growth factors including GCSF and GMCSF. Each of these were then applied to an immortalised human brain endothelial cell culture model system with barrier function measured using ECIS technology. Of all of the cytokines assessed, IL-1β and TNFα had the greatest effect on barrier strength. We observed a small transient reduction in barrier strength (in the first 10-14 hours), which was followed by a sustained strengthening by both IL-1β and TNFα (50-500pg/mL). IFNγ mediated a similar change in barrier strength. These responses were concentration dependent with IL-1β being the most potent. In contrast, IFNα caused the immediate strengthening of the barrier, whereas both IL6 and IL4 caused progressive weakening. Even though these cytokines influenced barrier function in different ways as demonstrated by the temporal power of ECIS technology, they each stimulated the brain endothelial cells causing increased secretion of various pro-inflammatory cytokines and chemokines. Most of the other factors measured in the sera from the MS patients did not have any influence on the barrier function or viability of the brain endothelial cells used in this study. We conclude that human brain endothelial cells are sensitive to a range of pleiotropic cytokines that are elevated in the sera of patients with RRMS. It is quite plausible that some or a combination of these may influence the BBB integrity and contribute to the leakiness occurring in regions of the brain susceptible to the formation of lesions.

Research Support: Neurological Foundation of New Zealand, and the University of Auckland Faculty Research Development Fund.

## A30 The impact of brain metastasis on endothelial cell function

### Christina Simoglou Karali^1,3^, Vinton Cheng^1^, Niloufar Zarghami^1^, Manuel Sarmiento Soto^1^, Yvonne Couch^2^, Daniel C.Anthony^3^ and Nicola R. Sibson^1^

#### ^1^Cancer Research UK & Medical Research Council Oxford Institute for Radiation Oncology, Department of Oncology, Oxford, UK, ^2^Radcliffe Department of Medicine (RDM), University of Oxford, Oxford, UK, ^3^Department of Pharmacology, University of Oxford, Oxford, UK

##### **Correspondence:** Christina Simoglou Karali (christina.simogloukarali@oncology.ox.ac.uk)

*Fluids and Barriers of the CNS* 2017, **14(Supp 2)**:A30

The presence of an intact, impermeable blood–brain barrier (BBB) during the early (micrometastatic) stages of tumour cell colonisation significantly limits early detection and effective treatment of brain metastasis. Systemic administration of tumour necrosis factor (TNF) in murine models transiently permeabilises the BBB selectively at sites of micrometastasis in the brain. This phenomenon facilitates enhanced detection of metastatic colonies with contrast-enhanced MRI and improved delivery of therapeutically-relevant molecules to the tumour site [1].

The aim of this study was to determine why tumour-associated endothelium becomes susceptible to TNF-induced BBB permeabilisation.

Immortalized human brain microvascular endothelial cells (hCMEC/D3) were exposed to the supernatants of human brain-seeking metastatic cancer cells (breast, MDAMB231Br; lung, SEBTA001; or melanoma, H1DL2), non-brain-targeting cancer cells (MDAMB231), cytokines (human TNF, huTNF; or a novel TNFR1-selective mutein, mutTNF) and TNFR1 siRNA. Expression of the endogenous receptors for TNF (TNFR1, TNFR2) was evaluated by qPCR and immunostaining. Alterations in paracellular permeability were measured using fluorescent dextrans.

The hCMEC/D3 monolayer showed significantly increased TNFR1 expression when stimulated with either brain-trophic tumour cells or tumour-conditioned media (*ca.* 120-fold for breast, 280-fold for lung, 260-fold for melanoma, p<0.001). In contrast, hCMEC/D3 TNFR2 expression was minimal on stimulation with breast or lung metastatic cells, and only modestly increased for melanoma cells (*ca.* 97-fold, p<0.001). No effect of non-brain targeting cancer cells on expression of either receptor was evident. Importantly, hCMEC/D3 treatment with cytokines, after stimulation of TNFR1 expression with tumour conditioned media from metastatic breast cancer cells, significantly increased permeability (*ca.* 15-fold for huTNF, 11-fold for mutTNF, p<0.001), whereas similar treatment on TNFR1-silenced, stimulated hCMECs did not exert any significant alterations in permeability.

Histological evaluation of tumours revealed that 55-80% (depending on tumour type) of vessels were TNFR1 positive up to a distance of 0.001- 0.099mm from the tumour boundary and 15-30% of vessels at a distance of 0.1-1mm from the tumour . Less than 10% of vessels were TNFR2 positive in all models.

These data confirm the specific upregulation of TNFR1 on tumour-activated endothelial cells both in vitro and in vivo within the tumour microenvironment. This study reveals that brain-seeking metastatic tumours secrete factors that alter normal brain endothelial cell function and may, in turn, enable the permeabilising effect of TNF on the BBB.

1. Connell JJ, Chatain G, Cornelissen B, Vallis KA, Hamilton A, Seymour L, Anthony DC, Sibson NR. Selective permeabilization of the blood–brain barrier at sites of metastasis. J Natl Cancer Inst. 2013; 105: 1634–43.

## A31 Unifying the neurovascular unit: Neurophysiology, behaviour and the blood–brain barrier

### John Kealy^1^

#### ^1^School of Biochemistry and Immunology, Trinity Biomedical Sciences Institute, Trinity College Dublin, Dublin 2, Ireland.

##### **Correspondence:** John Kealy (kealyjo@tcd.ie)

*Fluids and Barriers of the CNS* 2017, **14(Supp 2)**:A31

While it is accepted that the neuron is the fundamental cell of the nervous system, it is increasingly appreciated that neurons work in concert with numerous other cell types including glia, pericytes, and endothelial cells as a neurovascular unit (NVU). The components of the NVU act together to ensure that neurons are able to respond quickly and efficiently to changes in input. This is achieved through control of metabolism, removal of waste, regulation of neurotransmitters, and responding to immunological and inflammatory insults. Though we know much about the molecular and physiological functions of individual parts of the NVU, there is less known about how the NVU directly affects behaviour. My aim is to study NVU function across its various components in relation to behaviour. Using RNA interference, suppression of blood–brain barrier (BBB) tight junction proteins occludin and claudin-5 provide insights on to the effects of BBB permeability on behaviour with claudin-5 suppression in particular showing a marked effect on learning and memory, anxiety, and social behaviour. Targeted claudin-5 suppression reveals region-specific effects on behaviour. Importantly, long-term claudin-5 suppression may provide a potential to understand not only normal brain function but also the progression of numerous neurological disorders. Beyond the BBB, implantable amperometric sensors allow for simultaneous measurements of changes in neurochemistry along with local field potential in freely-moving rodents—allowing for direct comparison between neuronal function and NVU activity. This allows for crucial insights into how supply and utilisation of metabolic substrates by the NVU relates to changes in behaviour.

Grant support: Science Foundation Ireland (12/ TIDA/I2308; 12/YI/B2614; 11/PI/1080); Irish Research Council (GOIPD/2013/420); BrightFocus Foundation (A2015548S).

## A32 Endogenous mechanisms involved in barrier protection

### Richard F. Keep^1^, Lisa J. Routhe^2^, Jianming Xiang^1^, Hong Ye^1^, Ya Hua^1^, Torben Moos^2^, Guohua Xi^1^

#### ^1^Department of Neurosurgery, University of Michigan, Ann Arbor, MI, USA, ^2^Department of Health Science and Technology, Aalborg University, Aalborg, Denmark

##### **Correspondence:** Richard F. Keep (rkeep@umich.edu)

*Fluids and Barriers of the CNS* 2017, **14(Supp 2)**:A32

The brain, including the blood–brain and the blood-CSF (choroid plexus) barriers, has a range of mechanisms that are induced by and limit injury. For example, intraventricular hemorrhage, a devastating component of germinal matrix hemorrhage in premature infants and intracerebral hemorrhage in adults, upregulates iron handling proteins at the choroid plexus. Hemoglobin-derived iron is a major cause of injury after cerebral hemorrhage and the induction of such proteins may limit choroid plexus injury. There is also an upregulation of proteins involved in CSF secretion at the choroid plexus and increased CSF secretion might help clear the intraventricular hematoma. However, if the CSF flow pathway is blocked, it may also lead to hydrocephalus. While endogenous protective mechanisms can be induced after an injury, they can also be induced prior to injury by preconditioning stimuli. Preconditioning stimuli at the blood–brain and blood-CSF barriers include brief periods of ischemia and pharmacological agents such as isothiocyanates. Isothiocyanates, by inducing oxidative stress, activate the transcription factor nrf2, a master regulator of anti-oxidant defense mechanisms and thus induce many different proteins. The impact of current pharmacological strategies for treating stroke on endogenous neuroprotective mechanisms has received little attention. For example, do exogenous anti-oxidants prevent the activation of nrf2 and the upregulation of endogenous protective proteins? As endogenous neuroprotection involves multiple interlinked pathways, any negative impact of exogenous neuroprotectants on endogenous protection may serve to limit their effectiveness. Such effects may have contributed to the failure of many neuroprotectants that have gone into clinical trial for stroke.

## A33 The cell-penetrating peptide Tat facilitates delivery of a conjugated peptide drug across the blood–brain barrier

### M. Kristensen^1^, A. Bach^2^, K. Strømgaard^2^, B. Brodin^1^

#### ^1^Department of Pharmacy, Faculty of Health and Medical Sciences, University of Copenhagen, Copenhagen, Denmark, ^2^Department of Drug Design and Pharmacology, Faculty of Health and Medical Sciences, University of Copenhagen, Copenhagen, Denmark

##### **Correspondence:** M. Kristensen (mie.kristensen@sund.ku.dk)

*Fluids and Barriers of the CNS* 2017, **14(Supp 2)**:A33

A number of peptide drugs for the treatment of brain diseases are available. However, to reach their target site of action, they must pass the blood–brain barrier (BBB). The capillary endothelium comprises the major physical barrier of the BBB and allows only passive permeation of molecules <400 Da. Brain delivery of the larger biopharmaceuticals, which today includes an increasing number of novel peptide-drug entities, is therefore restricted; both due to their large molecular size and hydrophilic nature. Thus, the development of peptide-drugs for the treatment of brain specific diseases requires a delivery strategy for overcoming the endothelial BBB in order to reach its final target within the brain.

The cell-penetrating peptides (CPPs) comprise a promising tool to facilitate delivery of macromolecular drug entities not only into cells but also across biological barriers, such as the BBB. The CPP Tat, an 11-residue cationic peptide [1], has demonstrated potential as vector for brain delivery of the PSD-95 inhibitor NR2B9c, which hinders cell death following a stroke [2]. However, the mechanism by which Tat facilitates translocation of conjugated NR2B9c across the endothelial BBB is not known, and understanding the cellular events leading to Tat-membrane translocation likely lead to future development of optimized CPP-peptide drug constructs.

In the present study the delivery propensity of Tat was assessed using a primary bovine BBB model [3] and the porcine epithelial IPEC J2 cell line displaying transepithelial electrical resistance values (>1000 Ω*cm^2^) comparable to the that of a tight BBB. Tat significantly improved BBB translocation of NR2B9c across both the primary bovine BBB model as well as the IPEC J2 cell line. In order to explain the overall mechanism by which Tat facilitates NR2B9c barrier translocation, being via endocytic uptake or direct membrane translocation, transport studies were conducted at temperatures <37°C (25°C, 4°C) or in the presence of sodium acid, which hinders energy-dependent endocytic uptake. Neither at low temperature nor in the presence of sodium acid Tat mediated transport of NR2B9c across the IPEC-J2 cell culture model; thus demonstrating endocytic uptake being involved. In addition, heparin pre-incubation, which binds heparan sulphates (HS) at the cell surface, hindered transport of the Tat-NR2B9c conjugate, therefore indicating that the initial binding of Tat to HS may be important for mediating endocytic uptake.

1. Vivès E, Brodin P, Lebleu B. A truncated HIV-1 Tat protein basic domain rapidly translocates through the plasma membrane and accumulates in the cell nucleus. J Biol Chem. 1997;272: 16010–17.
2. Aarts M. Treatment of Ischemic Brain Damage by Perturbing NMDA Receptor- PSD-95 Protein Interactions. Science. 2002;298: 846–50.
3. Hans C Helms BB. Generation of primary cultures of bovine brain endothelial cells and setup of cocultures with rat astrocytes. Methods Mol Biol. 2014;1135: 205–11.

## A34 Two-photon microscopy of cerebral capillaries

### Martin Lauritzen^1, 2^, Mette Mathiesen Janiurek^1^, Nikolay Kutuzov^1^, Krzysztof Kucharz^1^

#### ^1^Department of Neuroscience, University of Copenhagen, Copenhagen, Denmark, ^2^Department of Neurophysiology, Rigshospitalet, Glostrup, Denmark

##### **Correspondence:** Martin Lauritzen (martin.johannes.lauritzen@regionh.dk)

*Fluids and Barriers of the CNS* 2017, **14(Supp 2)**:A34

The association of brain microvessels, astrocytes, pericytes, and neurons constitutes the neurovascular unit (NVU), and the interplay between these cell types controls the properties of the blood–brain barrier (BBB). Brain capillaries have traditionally been viewed as tubes that passively conduct blood from the heart to active nerve cells, but recent studies suggest that mural cells on brain capillaries are much more active than previously thought. First order capillaries are the first to dilate during neuronal activity and pericytes constrict capillaries in cerebral ischemia, which is expected to promote brain damage. However, the physiology of pericytes and brain capillaries is not completely understood and a number of important questions remain unanswered. For example, we know little about how capillaries communicate with one another to coordinate a blood flow response and the BBB tight and how this control affects the transport of nutrients and drugs across the BBB. This talk will summarize novel findings using two-photon microscopy in vivo on how capillary flow and permeability is controlled, how can it be quantified repeatedly in single and multiple capillaries in living mice.

## A35 Molecular control of the brain barriers in multiple sclerosis

### Melissa A. Lopes-Pinheiro^2^, Jamie Lim^2^, Alwin Kamermans^2^, Jack van Horssen^2^, Wendy W.J. Unger^1^, Ruud Fontijn^2^, Helga E. de Vries^2^

#### ^1^Department of Pediatrics, ErasmusMC, Rotterdam, The Netherlands, ^2^Department of Molecular Cell Biology and Immunology, VUmc MS Center Amsterdam, VU medical center, Amsterdam, The Netherlands

##### **Correspondence:** Helga E. de Vries (he.devries@vumc.nl)

*Fluids and Barriers of the CNS* 2017, **14(Supp 2)**:A35

Dysfunction of the neuroprotective blood–brain barrier (BBB) and blood-cerebrospinal fluid barrier (BCSFB) and inflammation thereof are key events in multiple sclerosis (MS). In early stages of MS, inflammation of the brain barriers actively aids in immune cell influx, inducing neuro-inflammation and demyelination. Upon MS progression, chronic BBB dysfunction is apparent, which contributes to gliosis and neurodegeneration. Moreover, barrier dysfunction goes hand in hand with a profound inflammation of the endothelial cells, thereby promoting infiltration of auto-reactive T-cells and monocytes, which further induces irreversible tissue damage. In turn, inflamed brain endothelium contributes to disease progression by its release of pro-inflammatory factors which activates the surrounding glia cells, together reinforcing astrogliosis, microglia activation and consequently neurodegeneration.

Our recent work suggests that under inflammatory conditions, brain endothelial cells (BECs) stimulate the migration of myelin-reactive T-cells by acting as non-professional antigen presenting cells through the processing and presentation of myelin-derived antigens in MHC-II. Inflamed BECs internalized myelin which was routed to endo-lysosomal compartment for processing in a time-dependent manner and subsequently induce T-cell activation and migration. In the transmigration of leukocytes across inflamed BECs, the enzyme acid sphingomyelinase (ASM), involved in the production of bio-active lipids such as ceramide, is essential for the coordination of the function of the endothelial adhesion molecule ICAM-1. These results demonstrate that BECs are capable of enhancing antigen-specific T cell recruitment. Moreover, current treatment regimens for MS, such as dimethylfumarate, are capable of reducing immune cell trafficking through the induction of endogenous anti-oxidant enzyme systems.

Together this illustrates that identification of the pathways that counteract BBB inflammation and reinstate proper BBB function will provide new and selective means to fight MS in different stages of disease.

Work was funded through grants of the Dutch MS research foundation

1. Lim JL, van der Pol SM, Di Dio F, van Het Hof B, Kooij G, de Vries HE, van Horssen J. Protective effects of monomethyl fumarate at the inflamed blood–brain barrier. Microvasc Res. 2016;105: 61–9.
2. Lopes Pinheiro MA, Kamermans A, Garcia-Vallejo JJ, et al. Internalization and presentation of myelin antigens by the brain endothelium guides antigen-specific T cell migration. Westbrook GL, ed. eLife. 2016;5: e13149.
3. Lopes Pinheiro MA, Kroon J, Hoogenboezem M, Geerts D, van het Hof B, van der Pol SMA, van Buul JD and de Vries HE. Acid Sphingomyelinase–Derived Ceramide Regulates ICAM-1 Function during T Cell Transmigration across Brain Endothelial Cells. J Immunol. 2016;196: 72–9.

## A36 Cerebrovascular inflammation in Guam Parkinsonism Dementia

### Petra Majerova^1^, Ralph M. Garruto^2^, Andrej Kovac^1^

#### ^1^Institute of Neuroimmunology, Slovak Academy of Sciences, Bratislava, Slovak Republic, ^2^Graduate Program in Biomedical Anthropology, Departments of Anthropology and Biological Sciences, Binghamton University, Binghamton, NY, USA

##### **Correspondence:** Petra Majerova (petra.majerova@savba.sk)

*Fluids and Barriers of the CNS* 2017, **14(Supp 2)**:A36

Parkinsonism-dementia complex (PDC) is a neurodegenerative disease with Parkinsonism and early-onset Alzheimer-like dementia. PDC belongs to the family of neurodegenerative disorders known as tauopathies that are histopathologically characterized by abnormal deposition of microtubule-associated protein tau. While changes in the blood–brain barrier (BBB) in Alzheimer’s disease are increasingly recognized, dysfunction of BBB in PDC has not been extensively studied. In present work we characterized cerebrovascular changes in patients with PDC.

Brain tissue from 10 postmortem PDC patients and 7 non-demented controls were assessed for structural and functional changes of BBB. Entorhinal cortex sections were immunostained for markers of brain endothelial cells (claudin-5, occludin and collagen IV) and inflammation (VCAM-1, ICAM-1, P-Selectin, E-Selectin). The ultrastructure of brain capillaries was investigated by confocal microscopy and morphological changes and intensity alterations were evaluated. We found significant decrease of tight junction proteins and upregulation of adhesion molecules that correlates with the presence of neurofibrillary tangles. Additionally, we showed presence of CD3^+^ and CD4^+^ positive cells in brain areas affected by pathological lesions. Our findings indicate that pathological lesions in PDC are associated with inflammatory changes of brain capillaries and could mediate transmigration of cells to brain parenchyma.

This work was supported by APVV-14-0547 and VEGA 2/0159/15.

## A37 Cellular and molecular mechanisms directing the pathway of Th1 versus Th17 T-cell diapedesis across the blood–brain barrier

### Luca Marchetti^1^, David Francisco^2^, Isabelle Gruber^1^, Ruth Lyck^1^, Rémy Bruggmann^2^ and Britta Engelhardt^1^

#### ^1^Theodor Kocher Institute, University of Bern, Bern, Switzerland, ^2^Interfaculty Bioinformatics Unit, University of Bern, Bern, Switzerland

##### **Correspondence:** Luca Marchetti (luca.marchetti@tki.unibe.ch)

*Fluids and Barriers of the CNS* 2017, **14(Supp 2)**:A37

The endothelial blood–brain barrier (BBB) maintains central nervous system (CNS) homeostasis and strictly controls T-cell trafficking into the CNS. During multiple sclerosis (MS) or its animal model, experimental autoimmune encephalomyelitis (EAE), autoaggressive γ-interferon-producing CD4^+^ Th1 or IL-17-producing CD4^+^ Th17 enter the CNS causing neuroinflammation. The molecular mechanisms mediating Th1 cells migration across the BBB are quite well studied, and we could previously show that endothelial cell surface levels of ICAM-1 direct CD4^+^ T cell to paracellular or transcellular sites for diapedesis. It has been suggested that Th17 cells may use different mechanisms from Th1 cells to extravasate. This prompted us to directly compare the cellular and molecular mechanisms used by those two CD4^+^ T-cells subsets to cross the BBB.

Using primary mouse brain microvascular endothelial cells (pMBMECs) as an in vitro BBB model, we compared the multi-step extravasation of in vitro polarized myelin oligodendrocyte (MOG) transgenic T-cell receptor Th1 and Th17 cells by live cell imaging under physiological flow. Our preliminary observations show higher numbers of Th1 than Th17 cells arrested on pMBMECs under noninflammatory and inflammatory conditions, whereas Th17 cells have higher tendency to detach. After subsequent polarization, Th1 cells crawled with higher speed over the pMBMECs compared to Th17 cells. Similar numbers of diapedesis events were observed for both subsets. It remains to be shown if Th1 and Th17 cells cross the BBB preferentially via the paracellular or transcellular route. Thus, we have demonstrated that the dynamic interaction of Th1 cells with the BBB is distinguishable from that of Th17 cells. To analyse their potential molecular mechanism, we are currently comparing the trafficking molecule signature of both T-cell subsets by multi-parameter flow cytometry. To identify genes involved in regulating the cellular pathway of T-cell diapedesis across the BBB, we have performed RNAseq analysis from pMBMECs expressing high or low levels of endothelial ICAM-1 and thus favouring trans- versus paracellular T-cell diapedesis, respectively. After sequential selections, we have identified a set of candidate genes for biological validation.

Identification of distinct molecular mechanisms mediating Th1 and Th17 cells’ migration across the BBB will allow us to accurately foresee CNS-specific adverse effects of the increasing numbers of therapies in many chronic inflammatory diseases targeting T-cell trafficking or even depleting peripheral T cells. Furthermore, our approach will allow us to identify novel therapeutic targets within the successful framework of therapeutic targeting of immune cell trafficking to the CNS for the MS treatment.

## A38 Targeted delivery of vesicular nanoparticles across a culture model of the blood–brain barrier

### Mária Mészáros^1^, Gergő Porkoláb^1^, Lóránd Kiss^1^, Ana-Maria Pilbat^2^, Zsolt Török^2^, Zsolt Bozsó^3^, Lívia Fülöp^3^, Mária A. Deli^1^ and Szilvia Veszelka^1^

#### ^1^Institute of Biophysics, Biological Research Centre, Hungarian Academy of Sciences, Szeged, Hungary, ^2^Institute of Biochemistry, Biological Research Centre, Hungarian Academy of Sciences, Szeged, Hungary, ^3^Department of Medical Chemistry, University of Szeged, Szeged, Hungary

##### **Correspondence:** Mária Mészáros (meszaros.maria@brc.mta.hu)

*Fluids and Barriers of the CNS* 2017, **14(Supp 2)**:A38

Efficient drug delivery across central nervous system (CNS) barriers is a central problem in pharmaceutical treatment of neurological diseases. Most pharmaceutical drug candidates including hydrophilic molecules, biopharmaceuticals, and efflux transporter ligands have a low permeability across the blood–brain barrier. Targeted nanoparticles are new tools to increase drug delivery to brain. The aim of our study was to compare different ligands of transporters present at the blood–brain barrier for targeting vesicular nanoparticles. Non-ionic surfactant and cholesterol-based nanoparticles, so-called niosomes were derivatized with alanin and glutathione using lipid or PEG linkers. The cargo of the niosomes was Evans blue-albumin complex. Treatments with niosomes did not influence the viability of primary rat brain endothelial cells. The presence of targeting ligands on niosomes increased the uptake of the cargo molecule in cultured brain endothelial cells which could be visualized by confocal microscopy. The cellular uptake of niosomes was temperature dependent and could be decreased with sodium azide, a metabolic inhibitor indicating an active transport process. Filipin and cytochalasin D, inhibitors of endocytosis decreased the uptake of the targeted nanoparticles in brain endothelial cells. The modifications of the charge of the endothelial glycocalix with TMA-DPH (1- (1-(4-trimethylammoniumphenyl)-6-phenyl-1,3,5-hexatriene p-toluenesulfonate) or removing the glycocalyx with neuraminidase elevated the uptake of the targeted nanocarriers. Targeting ligands enhanced the permeability of the cargo across brain endothelial monolayers. Treatment with niosomes increased plasma membrane fluidity in endothelial cells, suggesting the fusion of the nanovesicles with the cell membranes. Our data indicate that nutrient transporter ligands can potentially be exploited for CNS targeting of nanoparticles.

Supported by grants from the Hungarian Scientific Research Fund (OTKA PD105622), and the National Research, Development and Innovation Office (GINOP-2.2.1-15-2016-00007, GINOP-2.3.2-15-2016-00060). S.V. was supported by the János Bolyai Research Fellowship of the Hungarian Academy of Sciences (BO/00724/12). G.P. is a Szent-Györgyi student in the Szeged Scientists Academy Program of the Foundation for the Future of Biomedical Sciences in Szeged implemented with the support of the Ministry of Human Resources (TSZ:34232-3/2016/INTFIN).

## A39 Determination of the blood–brain barrier integrity in transgenic rat model for tauopathies SHR-24 using evans blue and sodium fluorescein

### Alena Michalicova^1,2^, Jaroslav Galba^2,4^, Sandra Mihaljevic^1,2^, Michal Novak^1,2^, and Andrej Kovac^1,2,3^

#### ^1^Institute of Neuroimmunology, Slovak Academy of Sciences, Bratislava, Slovak Republic, ^2^ AXON Neuroscience R&D Services SE, Bratislava, Slovak Republic, ^3^ Department of Pharmacology and Toxicology, University of Veterinary Medicine and Pharmacy, Kosice, Slovak Republic, ^4^ Faculty of Pharmacy of Comenius University, Department of Pharmaceutical Analysis and Nuclear Pharmacy, Bratislava, Slovak Republic

##### **Correspondence:** Alena Michalicova (alena.michalicova@savba.sk)

*Fluids and Barriers of the CNS* 2017, **14(Supp 2)**:A39

The blood–brain barrier (BBB) is formed by a layer of endothelial cells that line cerebral microvessels. Neighbouring endothelial cells are strongly attached to each other by tight junctions, which represent a physical barrier between the brain parenchyma and blood circulation. The integrity of the BBB ensures the ionic homeostasis necessary for the neuronal activity, correct flow of nutrients from the blood to the brain and prevents the entry of potentially harmful substances to the CNS. It is known, that the BBB changes are an integral part of many neurodegenerative diseases, including tauopathies. Tauopathies represent a heterogeneous group of neurodegenerative diseases characterized by an abnormal deposition of microtubule-associated protein tau in cells of the central nervous system, such as Alzheimer´s disease, Down´s syndrome, progressive supranuclear palsy, Pick´s disease, corticobasal degeneration, frontotemporal dementia with Parkinsonism linked to chromosome-17, traumatic brain injury and others. To demonstrate structural and functional changes of the BBB, suitable models and markers are required. Sodium fluorescein (SF) and Evans blue (EB) represent the most commonly used integrity tracers, despite their limitations. In our study, we used SF as a marker for small molecules and EB for plasma proteins. While EB binds to plasma proteins to become a high molecular weight protein tracer, SF remains unbound in the blood stream. The concentration of SF in plasma and brain tissue samples was measured using Fluoroscan Ascent FL (485 nm/538 nm) and to monitor the extravasation of albumin across the BBB, we developed an ultrahigh-performance liquid chromatography method with UV detection (605 nm). Using these methods, we analysed the integrity of the BBB in transgenic rat model for tauopathies SHR-24 expressing human truncated tau (151-391) and age-matched control animals.

**Acknowledgements:** The authors would like to thank Axon Neuroscience for providing the transgenic animals for this study. This work was supported by competitive academic grants VEGA 2/0159/15 and APVV-14-0547 and structural funds 26240220008, 26240220046.

## A40 Analysis of the changes on the choroid plexus barrier in tauopathies

### Sandra Mihaljevic

#### Institute of Neuroimmunology, Slovak Academy of Sciences, Bratislava, Slovak Republic

##### **Correspondence:** Sandra Mihaljevic (sandra.mihaljevic@gmail.com)

*Fluids and Barriers of the CNS* 2017, **14(Supp 2)**:A40

Alzheimer´s disease (AD) is a chronic neurodegenerative disease that damages the higher brain functions. Control of traffic of different substances between CNS and blood circulation is controlled by three barriers: arachnoid epithelium, blood–brain barrier (BBB) and choroid plexus epithelium (BCSFB). They represent highly dynamic system of cells and are involved in optimal brain functioning, synaptic transmission, synaptic remodeling, angiogenesis, neurogenesis etc. In case that barrier’s integrity is disrupted on any mode, many of vital brain functions may be endangered. Today, we know that brain is not “immune privileged” and that many pathological states are involved in BBB breakdown and disorders, including AD. Better understanding of the anatomy and cell biology of these neurovascular units in health and disease is critical for advancement of therapeutic development.

While BBB being extensively studied, BCSFB has been largely understudied, but there are indications that its role is crucial in maintaining brain homeostasis, and even has a compensatory effect in case of BBB malfunctioning.

In our work we are focused on role of BCSFB in neurodegeneration. We showed presence of tau protein in epithelial cells of choroid plexus in animal model for tauopathy. We analyzed expression of transport proteins: LRP-1 and RAGE and showed changes in expression of pro-inflammatory epithelial molecules. A thickening of epithelial basal membrane and greater collagen- IV depositions occurred around capillaries in choroid plexus. These findings indicate structural and functional changes of choroid plexus in animal model for tauopathies.

This work was supported by competitive academic grants VEGA 2/0159/15 and APVV-14-0547 and structural funds 26240220008, 26240220046.

## A41 In vitro analysis to evaluate brain metastatic potential of cancer cells from human surgical specimens—preliminary report

### Yoichi Morofuji^1^, Takashi Fujimoto^1^, Daisuke Watanabe^2^, Shinsuke Nakagawa^2^, Kenta Ujifuku^1^, Nobutaka Horie^1^, Tsuyoshi Izumo^1^, Takeo Anda^1^, Takayuki Matsuo^1^

#### ^1^Department of Neurosurgery, Nagasaki University Graduate School of Biomedical Sciences, Nagasaki, Japan, ^2^Department of Medical Pharmacology, Nagasaki University Graduate School of Biomedical Sciences, Nagasaki, Japan

##### **Correspondence:** Yoichi Morofuji (yoichi51@hotmail.com)

*Fluids and Barriers of the CNS* 2017, **14(Supp 2)**:A41

Brain metastases are the most common brain neoplasms seen clinically in the adults and comprise more than half of all brain tumors. *Brain metastasis* presents an emerging and urgent unmet medical need and that has been historically understudied. To evaluate brain metastatic process, our group has developed in vitro blood–brain barrier (BBB) model and established the method of isolation and culture of primary cancer cells from human surgical specimens. Our model possesses the unique capability to examine brain metastases of human cancer cells and their therapeutic responses to chemotherapy. In our model, cancer cells were introduced to the luminal chamber. Cancer cells could pass through the BBB and drop onto the astrocytes in abluminal chamber. We observed the colony formation of cancer cells that expressed of specific tumor markers and reduction of transendothelial electrical resistance (TEER). The results also suggest that the interactions between cancer cells and astrocytes in BBB microenvironment might affect the proliferation of cancer cells. Such primary human cancer cell cultures can serve as a model of choice for the study of the mechanisms behind key aspects of brain metastases biology, including metastatic process and drug testing. The goal of this study is to establish personalized treatment protocol for brain metastases based on cell culture.

Grant support: This work was supported by JSPS KAKENHI Grant number 17K10840 and 15KK0349

## A42 Cocaine-mediated secretion of IP-10 from pericytes: implication for monocyte recruitment into the CNS

### Fang Niu, Shilpa Buch

#### Department of Pharmacology and Experimental Neuroscience, University of Nebraska Medical Center, Omaha, NE, USA

##### **Correspondence:** Shilpa Buch (sbuch@unmc.edu)

*Fluids and Barriers of the CNS* 2017, **14(Supp 2)**:A42

Drug abuse and addiction have been closely linked with HIV / AIDS because the infection easily spreads among drug abusers. Epidemiological studies on drug abusers with AIDS link cocaine abuse, more than other drugs, to acceleration of HIV infection and the progression of NeuroAIDS. Cocaine has been known to facilitate the transmigration of inflammatory leukocytes into the brain, which is an important mechanism for HIV entry into the central nervous system (CNS). Pericytes, as important constituents of the blood–brain barrier (BBB), play a key role in maintaining the BBB integrity yet remain poorly studied in the context of CNS inflammation. In the present study, we demonstrate that human brain vascular pericytes (HBVPs) exposed to cocaine manifest increased secretion of the chemokine IP-10, with a concomitant induction of monocyte transmigration across BBB both in vitro and in vivo. Furthermore, our findings demonstrated a time-dependent translocation of NF-kB in HBVPs after cocaine-stimulation. Further dissection of NF-κB regulation using both the pharmacological and genetic approaches revealed that the NF-κB nuclear translocation was dependent on σ-1R, c-Src and PDGFR-β signal activation. Finally, our study demonstrated that IP-10 release was mediated by activation and translocation of sigma-1 receptor (σ-1R) and subsequent interaction of σ-1R with c-Src kinase leading to activation of the Src-PDGFR-β-NF-κB pathway and up-regulation of IP-10 expression. These findings implicate a novel role of IP-10 in the crosstalk of pericytes and monocytes in cocaine-mediated neuroinflammation in the CNS, further underscoring the role of pericytes as active components of the innate immune responses.

**Keywords:** IP-10, pericytes, cocaine, monocyte transmigration, sigma-1 receptor, blood–brain barrier

**Acknowledgements:** This work was supported by grants DA040397 from the National Institutes of Health.

## A43. Expression of pattern recognition receptors and activation of the non-canonical inflammasome pathway in brain pericytes

### Ádám Nyúl-Tóth^1^, Mihály Kozma^1^, Péter Nagyőszi^1^, Krisztina Nagy^1^, Csilla Fazakas^1^, János Haskó^1^, Kinga Molnár^1^, Attila E. Farkas^1^, Péter Galajda^1^, Imola Wilhelm^1^, István A. Krizbai^1^

#### ^1^Institute of Biophysics, Biological Research Centre, Hungarian Academy of Sciences, Szeged, Hungary

##### **Correspondence:** István A. Krizbai (krizbai@brc.hu)

*Fluids and Barriers of the CNS* 2017, **14(Supp 2)**:A43

Cerebral pericytes are mural cells embedded in the basement membrane of capillaries. Increasing evidence suggests that they play important role in controlling neurovascular functions, i.e. cerebral blood flow, angiogenesis and permeability of the blood–brain barrier. These cells can also influence neuroinflammation which is highly regulated by the innate immune system. Therefore, we systematically tested the pattern recognition receptor expression of brain pericytes. We detected expression of NOD1, NOD2, NLRC5, NLRP1-3, NLRP5, NLRP9, NLRP10 and NLRX mRNA in non-treated cells. Among the ten known human TLRs, TLR2, TLR4, TLR5, TLR6 and TLR10 was found to be expressed. Inflammatory mediators induced the expression of NLRA, NLRC4 and TLR9 and increased the levels of NOD2, TLR2, inflammasome-forming caspases and inflammasome-cleaved interleukins. Oxidative stress, on the other hand, upregulated expression of TLR10 and NLRP9. Activation of selected pattern recognition receptors can lead to inflammasome assembly and caspase-dependent secretion of IL-1β. TNF-α and IFN-γ increased the levels of pro-IL-1β and pro-caspase-1 proteins; however, no canonical activation of NLRP1, NLRP2, NLRP3 or NLRC4 inflammasomes could be observed in human brain vascular pericytes. On the other hand, we could demonstrate secretion of active IL-1β in response to non-canonical inflammasome activation, i.e. intracellular LPS or infection with E. coli bacteria. Our results indicate that pericytes might have an important regulatory role in neuroinflammation.

Grant support: This work was supported by the National Research, Development and Innovation Office (grant numbers: OTKA K-116158, OTKA PD-121130, OTKA PD-112509, GINOP-2.3.2-15-2016-0020, GINOP-2.3.2-15-2016-0034, GINOP-2.3.2-15-2016-0030 and GINOP-2.3.2-15-2016-00001). Á.N-T. was supported by the Doctoral Scientific Scholarship of the Doctoral Institute of University of Szeged and by the ÚNKP-16-3/1. VI. 2. New National Excellence Program of the Ministry of Human Capacities. I.W. was supported by the János Bolyai Research Fellowship of the Hungarian Academy of Sciences (BO/00334/16/8).

## A44 Dynamic blood brain barrier regulation in sub-concussive brain injuries

### Eoin O’Keefe^1^, Eoin Kelly^2^, Eugene Wallace^2^, Chris Greene^1^, Stephanie Hughes^3^, John Kealy^1^, Niamh Doyle^3^, Marian M. Humphries^1^, Michael Farrell^4^, Gerald A. Grant^5^, Alon Friedman^6,7^, Ronel Veksler^6^, Michael G. Molloy^8^, James F. Meaney^9^, Niall Pender^3^, Colin P. Doherty^2^ and Matthew Campbell^1^

#### ^1^Smurfit Institute of Genetics, Trinity College Dublin, Dublin 2, Ireland., ^2^Department of Neurology, Health Care Centre, Hospital 5, St James’s Hospital, Dublin 8, Ireland., ^3^Department of Psychology, Beaumont Hospital, Dublin 9, Ireland, ^4^Department of Neuropathology, Beaumont Hospital, Dublin 9, Ireland., ^5^Department of Neurosurgery, Stanford University School of Medicine, Stanford, California, USA., ^6^Department of Cognitive and Brain Sciences, Zlotowski Center for Neuroscience, Ben-Gurion University of the Negev, Beer-Sheva, Israel., ^7^Department of Medical Neuroscience, Dalhousie University, Halifax, Canada., ^8^Department of Medicine, University College Cork, Cork, Ireland., ^9^Department of Radiology, St James’s Hospital, Dublin 8, Ireland.

##### **Correspondence:** Eoin O’Keefe (eookeeff@tcd.ie)

*Fluids and Barriers of the CNS* 2017, **14(Supp 2)**:A44

Traumatic brain injury (TBI) continues to be a global a health issue, contributing to the deaths of thousands and the debilitation of many more each year. While moderate and severe TBI may be recognised by overt symptoms or abnormalities on conventional imaging modalities (MRI or CT scans), injuries categorised as mild, injuries commonly referred to as concussions, rely on individual neuropsychological assessments. While undetected by current neuroimaging paradigms, subtle changes in the gliovascular unit that may following mild or sub-mild TBI may contribute to development of neurodegenerative conditions later in life, as suggested by findings of Blood Brain Barrier disruption (BBBD) in an individual diagnosed with Chronic Traumatic Encephalopathy, a condition primarily associated with repeated mild TBI.

Here we present evidence of changes at the Blood Brain Barrier in a sub-set of children following a season of Rugby Union, measured by Gadolinium extraversion signal. Paired with this were findings of increased levels of BDNF and MCP-1 in plasma samples, in addition to increased pro-inflammatory response by Peripheral Blood Mononuclear Cells post-season, as measured by IL- 1β production following necrotic brain tissue stimulation. In addition, examining a young adult cohort produced similar finding when examined within hours of engaged in a Rugby Union match. These finding suggest that a combination of analytical methods are required in order to gain an understanding of potential neural damage occurring due to mild TBI or sub-concussive blows.

Grant support: Science Foundation Ireland, Health Research Board of Ireland, BrightFocus Foundation, St. James’ Hospital Foundation.

## A45 Protective impact of exercise on methamphetamine-induced BBB disruption and aberrant neurogenesis in the hippocampus

### Minseon Park^1^, Arkadiusz Liskiewicz^2,3^, Marta Przybyla^2,4^, Daniela Kasprowska-Liśkiewicz^2^, Marta Nowacka-Chmielewska^2^, Andrzej Malecki^2^, Michal Toborek^1,2^

#### ^1^Department of Biochemistry and Molecular Biology, Miller School of Medicine, University of Miami, Miami, FL, USA, ^2^Jerzy Kukuczka Academy of Physical Education, Katowice, Poland, ^3^Department of Physiology, Medical University of Silesia, Katowice, Poland, ^4^Department for Experimental Medicine, Medical University of Silesia, Katowice, Poland

##### **Correspondence:** Michal Toborek (mtoborek@med.miami.edu)

*Fluids and Barriers of the CNS* 2017, **14(Supp 2)**:A45

There is no effective therapeutic intervention developed targeting cerebrovascular toxicity of drugs of abuse, including methamphetamine (METH). We hypothesize that exercise can protect against METH-induced neurotoxicity by enhancing the blood–brain barrier (BBB) integrity and protection against aberrant hippocampal neurogenesis. To address this notion, mice were administered with METH for 5 days with an escalating dose regimen, followed by voluntary wheel running for 2 weeks resembling the voluntary pattern of human exercise. The frequency, duration, and intensity of each running session were monitored for each mouse via a direct data link to a computer and the running data are analyzed by Clocklab™ Analysis software. Controls included sedentary mice that did not have access to running wheels and/or were injected with saline. METH administration disrupted hippocampal neurogenesis by inducing aberrant differentiation of neural progenitor cells to both immature and mature neurons. In addition, METH increased hippocampal BBB permeability by disrupting expression and localization of tight junction proteins. These changes were preserved in sedentary mice; however, exercise significantly attenuated METH-induced aberrant neurogenesis and disruption of the BBB integrity. Importantly, abnormal neurogenesis observed in METH-treated mice was associated with alternations of spatial memory as evaluated by Morris water maze. These behavioral changes were also attenuated by exercise. Overall, our results indicate that physical exercise can protect against METH-induced alterations of neurogenesis presumably by stabilization of the BBB.

Grant support: This work was supported by the NIH, grants DA039576, DA044579, HL126559, MH098891, MH072567, and by the NSC grant 2015/17/B/NZ7/02985.

## A46 Cellular interactions in the neurovascular unit during brain development

### Ana Pombero, Raquel Garcia-Lopez, Marta Martinez-Morga and Salvador Martinez

#### Instituto de Neurociencas de Alicante, Alicante, Spain

##### **Correspondence:** Salvador Martinez (smartinez@umh.es)

*Fluids and Barriers of the CNS* 2017, **14(Supp 2)**:A46

Neurogenesis and angiogenesis are processes that occur at the same time during brain development. Neurogenesis is a basic process occurring during embryonic development that proceeds throughout the adult life in the neurogenic areas, such as subventricular zone and the subgranular zone of the dentate gyrus (DG). In the DG the maintained neurogenesis represents a structural and functional singularity, which may determine its complex vascular pattern. Neurogenesis and angiogenesis share molecular signals and are mutually promotive, which support the concept of neurogenic niche in the brain, a functional unit between the precursor cells and their local microenvironment, in which blood vessel represent a key element. Whereas it is well-known that vascular development controls proliferation during brain development and in the adult by neurotrophic factors release, it still remains elusive how neural cells signal to vascular components during angiogenesis. We have demonstrated that the reduction of neural progenitor cells leads to an important impairment of the vascular development together with a reduction of the quantity of proliferating cells near blood vessels. Since VEGF is a potential regulator in neurogenesis and angiogenesis crosstalk, we are interested in assess the potential role of this molecule in the hippocampal neurovascular niche. Our results point out that neural progenitors secrete VEGF-A to regulate the vascular development in the dentate gyrus.

## A47 Neurovascular interactions under blood–brain barrier dysfunction

### Ofer Prager^1^, Lyna Solomon-Kamintsky^4^, Karl Schoknecht^2^, Guy Bar-Klein^3^, Dan Milikovsky^1^, Udi Vazana^1^, Dror Rosenbach^1^, Richard Kovács^2^, Alon Friedman^1,4^

#### ^1^Departments of Physiology & Cell Biology, Cognitive & Brain Sciences, the Zlotowski Center for Neuroscience, Ben-Gurion University of the Negev, Beer-Sheva, Israel, ^2^Institute for Neurophysiology, Charité—University Medicine Berlin, Berlin, Germany, ^3^Howard Hughes Medical Institute and Institute of Genetic Medicine, Johns Hopkins University School of Medicine, Baltimore, MD, USA, ^4^Department of Medical Neuroscience, Faculty of Medicine, Dalhousie University, Halifax, Canada

##### **Correspondence:** Alon Friedman (alonf@bgu.ac.il)

*Fluids and Barriers of the CNS* 2017, **14(Supp 2)**:A47

The microvascular endothelial cells in the brain, connected by tight junctions, are surrounded by pericytes, astrocytes, microglia and neurons. Together they act as a functional unit, named the neurovascular unit (NVU). Proper functioning of the NVU in general is critical for normal brain functions and homeostasis. Accumulated recent data from human and animal studies reveal that increased permeability of the blood–brain barrier (BBB) is a major determinant in common neurological disorders. We have previously shown that long-lasting dysfunction of the BBB leads to astrocytic transformation and epileptogenesis [1, 2, 3, 4]. Inversely, vascular injury and specifically BBB dysfunction are well-documented outcomes of epileptic seizures. However, the detailed endothelial pathology and mechanisms underlying increased permeability of the BBB are poorly understood. Here we will present recent data, directing for the role of neuronal-enhanced glutamate release and NMDA-receptors in seizure-induced opening of the BBB [5]. We will next describe our recent studies on the key role of NVU interactions in the regulation of oxygenated-blood supply to the metabolic demand of activated brain cells (“neurovascular-coupling”). As pericytes were described to have an important role in the integrity of the BBB, we will show that recurrent seizures are associated with pericytic injury and BBB dysfunction, and result with impaired vascular response to seizures at both capillary and arteriolar levels. Finally, we will discuss new data suggesting endothelial pathology as a diagnostic and treatment target for brain disorders [6, 7].

Grant support: European Union’s Seventh Framework Program (EU, FP7), DFG and EXC Neurocure grants. NIH Israel Science Foundation (ISF), German Science Foundation (DFG), the CURE Epilepsy Foundation, the Nova Scotia Health Research Foundation and Canada Institute for Health Research (CIHR).

1. Seiffert E, Dreier JP, Ivens S, Bechmann I, Tomkins O, Heinemann U, Friedman A. Lasting blood–brain barrier disruption induces epileptic focus in the rat somatosensory cortex. J Neurosci. 2004;24:7829–36.
2. Ivens S, Kaufer D, Flores LP, Bechmann I, Zumsteg D, Tomkins O, Seiffert E, Heinemann U, Friedman A. TGF-β receptor-mediated albumin uptake into astrocytes is involved in neocortical epileptogenesis. Brain. 130: 535–47.
3. Cacheaux LP, Ivens S, David Y, et al. Transcriptome Profiling Reveals TGF-β Signaling Involvement in Epileptogenesis. The Journal of neuroscience : the official journal of the Society for Neuroscience. 2009;29(28):892–35.
4. Bar-Klein G, Cacheaux LP, Kamintsky L, et al. Losartan prevents acquired epilepsy via TGF-β signaling suppression. Ann Neurol. 2014;75: 864–75.
5. Vazana U, Veksler R, Pell GS, et al. Glutamate-Mediated Blood–Brain Barrier Opening: Implications for Neuroprotection and Drug Delivery. J Neurosci. 2016;36: 7727–39.
6. Bar-Klein G, Klee R, Brandt C, Bankstahl M, Bascuñana P, Töllner K, Dalipaj H, Bankstahl JP, Friedman A, Löscher W. Isoflurane prevents acquired epilepsy in rat models of temporal lobe epilepsy. Ann Neurol. 2016;80: 896–908.
7. Bar-Klein G, Lublinsky S, Kamintsky L, Noyman I, Veksler R, Dalipaj H, Senatorov VV Jr, Swissa E, Rosenbach D, Elazary N, Milikovsky DZ, Milk N, Kassirer M, Rosman Y, Serlin Y, Eisenkraft A, Chassidim Y, Parmet Y, Kaufer D, Friedman A. Imaging blood–brain barrier dysfunction as a biomarker for epileptogenesis. Brain. 2017;140: 1692–1705

## A48 The neuroprotective role of physical exercise

### Zsolt Radak

#### University of Physical Education, Budapest, Hungary

##### **Correspondence:** Zsolt Radak (radak@tf.hu)

*Fluids and Barriers of the CNS* 2017, **14(Supp 2)**:A48

Regular exercise has systemic beneficial effects, including the promotion of brain function. The adaptive response to regular exercise involves the up-regulation of the enzymatic antioxidant system and modulation of oxidative damage. Reactive oxygen species (ROS) are important regulators of cell signaling. Exercise, via intensity-dependent modulation of metabolism and/or directly activated ROS generating enzymes, modulates the cellular redox state of the brain. ROS are also involved in the self-renewal and differentiation of neuronal stem cells and the exercise-mediated neurogenesis could be partly associated with ROS production. Regular physical exercise and nutritional intervention decrease both the incidence and symptom intensity of Alzheimer’s disease (AD) and modulate microbiome. When APP/PS1 mice were subjected to exercise and probiotics they significantly outperformed controls, whereas exercise, prebiotics alone and the two together resulted in decreases in beta-amyloid plaques, and increased microglia numbers around the plaques. Moreover, data also showed that exercise training increased the levels of anti-inflammatory microorganisms, such as bacteria that are involved in butyrogenesis. Overall, it is clear that physical exercise exerts neuroprotective effects on brain via complex mechanisms.

Exercise has strong effects on the immune system and readily alters the production of cytokines. Certain cytokines, especially IL-6, IL-1, TNF-a, IL-18 and IFN gamma, are actively involved in the modulation of synaptic plasticity and neurogenesis. Cytokines can also contribute to ROS production. ROS mediated alteration of lipids, protein, and DNA could directly affect brain function, while exercise modulates the accumulation of oxidative damage. Oxidative alteration of macromolecules can activate signaling processes, membrane remodeling, and gene transcription. The well-known neuroprotective effects of exercise are partly due to redox- associated adaptation.

## A49 The role of the choroid plexus in multiple sclerosis

### Sabela Rodríguez-Lorenzo^1^, David M. F. Francisco^2^, Remy Bruggmann^2^, Gijs Kooij^1^, Helga E. de Vries^1^

#### Department of Molecular Cell Biology and Immunology, VUmc MS center Amsterdam, Neuroscience Campus Amsterdam, VUmc, Netherlands, ^2^ Interfaculty Bioinformatics Unit, University of Bern and Swiss Institute of Bioinformatics, Bern, Switzerland

##### **Correspondence:** Helga E. de Vries (he.devries@vumc.nl)

*Fluids and Barriers of the CNS* 2017, **14(Supp 2)**:A49

The choroid plexi (CP) are highly vascularised projections of the brain ventricles that act as barriers between the blood and the cerebrospinal fluid (CSF), forming the blood-CSF-barrier (BCSFB). Their best known function is to produce CSF, but increasing evidence indicates CP are also involved in the maintenance of immune homeostasis of the central nervous system (CNS), thereby indirectly regulating immune cell entry into the CNS. Multiple sclerosis (MS) is an autoimmune demyelinating disease of the CNS characterized by chronic inflammation and neurodegeneration. We hypothesise that, due to its essential function, a dysregulation of the CP could contribute to MS pathology.

To reveal potential alterations in the CP associated with MS, we performed RNA-sequencing on human post-mortem CP derived from the lateral ventricles of 6 progressive MS patients and 6 non-neurological controls. Total RNA was isolated and only samples with good quality RNA (RIN > 7) were selected, enriched by rRNA depletion and used for paired-end sequencing.

Significant alterations in the CP transcriptome were identified which predominantly revealed pathways involved in cell migration, adhesion, inflammation, angiogenesis, and oxidative stress. We identified 14 genes differentially expressed (padj < 0.05, baseMean > 50) between the MS and the control CP.

Further validation and functional studies of the identified genes will shed light on the specific roles of the CP in MS. The identification of CP specific pathways contributing to MS pathogenesis will provide novel insights into the disease and may yield new targets for treatment.

Grant support: BtRAIN Marie-Curie Network, NAUTA fonds and VUmc MS Center Amsterdam

## A50 Sound as safe and cost-effective method for brain drug delivery

### Semyachkina-Glushkovskaya Oxana, Bragin Denis, Bragina Olga, Vodovozova Elena, Alekseeva Anna, Salmina Alla, Salmin Vladimir, Morgun Andrey, Malinovskaya Nataliya, Khilazheva Elena, Boytsova Elizaveta, Shirokov Alexander, Maslyakova Galina, Navolokin Nikita, Bucharskaya Alla, Yang Yirong, Abdurashitov Arkady, Gekalyuk Artem, Ulanova Mariya, Shushunova Natalia, Bodrova Anastasia, Sagatova Madina, Khorovodov Alexander, Shareef Ali Esmat, Pavlov Alexey, Tuchin Valery, Kurths Jürgen

#### Saratov State University, Interdisciplinary Center of Critical Technologies in Medicine, Department of Physiology of Human and Animals, Saratov, Russian Federation

##### **Correspondence:** Semyachkina-Glushkovskaya Oxana (glushkovskaya@mail.ru)

*Fluids and Barriers of the CNS* 2017, **14(Supp 2)**:A50

The motivation of our study is the actual problem in neurosciences as the development of methods for the brain drug delivery, which can open the door of progress to the therapy of central nervous systems diseases. There are more than 70 technologies for overcoming of the blood–brain barrier (BBB). However, no one of them is widely used in daily clinical practice due to invasiveness, limitation of treatment area or drug concentration and other complications.

This study is focused on the development of new non-invasive method for the reversible opening of BBB by sound (110 dB, 370 Hz, duration 2h), which is usual for daily life in modern big cities.

The independent ex vivo and in vivo results on mice obtained in twelve different research groups clearly demonstrate that sound significantly increases the BBB permeability to both high and low molecular weight substances as well as to liposomes (100 nm) that mimics the delivery into the brain solutes, proteins and nanocarrier materials. The physiologists, chemists, and physicists studied the effectiveness of sound to open the BBB and mechanisms underlying these process using classical and modern approaches in the assessment of the BBB permeability and cerebral microcirculation, such as confocal and magnetic resonance imaging, two photon microscopy, spectrofluorometric assay. The safety and reversibility of non-invasive effects of sound on the BBB permeability were confirmed by histological analysis of the brain tissues and vessels on the large groups of mice that as evaluated by qualified anatomists.

Thus, sound as natural factor due to its high effectiveness and easy execution has good perspective to be widely introduced in daily clinical practice for successful brain drug delivery.

This work was supported by Grant of Russian Science Foundation № 17-15-01263.

## A51 Toxicity and internalization of polymeric nanoparticles in brain endothelial cells

### Marcelle Silva de Abreu^1^, Ana C. Calpena^1^, Marta Espina^1^, Maria Luisa García^1^, Ignacio A. Romero ^2^, David Male ^2^

#### ^1^University of Barcelona, Faculty of Pharmacy and Food Sciences, Department of Pharmacy, Pharmaceutical Technology and Physicochemical, Barcelona, Catalonia, Spain, ^2^ School of Life Science, Health and Chemical Sciences, Faculty of Science, The Open University, Walton Hall, Milton Keynes, United Kingdom.

##### **Correspondence:** Marcelle Silva de Abreu (marcellesabreu@gmail.com)

*Fluids and Barriers of the CNS* 2017, **14(Supp 2)**:A51

The blood brain barrier (BBB) is a barrier that protects the brain against various harmful substances. However, it is an important limitation for the development of new drugs. For this reason, there is a constant search in find new alternatives to deliver drugs to the brain. In this way, polymeric nanoparticles (NPs) from (D,L-lactide-co-glycolide) poly(ethylene glycol) (PLGA-PEG) could be an important strategy to facilitate the transport of therapeutic molecules across the BBB; they are a drug delivery systems that is biodegradable, biocompatible and approved by Food and Drug Administration (FDA). The main objective of this work was the encapsulation of an anti-diabetic drug in polymeric NPs of PLGA-PEG to analyze its toxicity and internalization using a human brain microvascular endothelial cell line (hCMEC/D3). The NPs were prepared by the solvent displacement technique. An initial study used a factorial design to modify the independent variables determining NP synthesis. Subsequently, physicochemical, biopharmaceutical and stability characterization studies were done. To evaluate the toxicity and uptake, NPs, at concentrations up to 10 µg/mL, were applied to the hCMEC/D3 cell line. Uptake was measured by flow cytometry (FACSCalibur) with rhodamine-labelled NPs. The experiment was standardized to 10,000 events on the gate and at voltage of 410 mV with respect to the control sample (untreated cells). The results demonstrated that the NPs (mean size around 160 nm and negative charge of −14.4 mV), were internalized by the cells, and showed the character of a monodisperse systems. The release profile showed a slow release with a Fick’s passive diffusion. In the toxicity assay, cells were 80% viable up to a concentration of 5 µg/mL. Fluorescence and electron microscopy indicated that NPs were primarily present in the cells’ cytosol. Taken together, the results demonstrate that these nanoparticles could be effective in delivering therapeutics to the central nervous system.

Grant support: The authors would like to thank CAPES (Coordination for the Improvement of Higher Education Personnel) the Brazilian and the Spanish Ministries of Science and Innovation grant (MAT2014-59134R) and Sheffield Hospitals Trust for financial support.

## A52 Clearance mechanisms of amylogenic proteins across the blood brain barrier

### Steffen Storck^1^, Anika Hartz^2^, Jens Pahnke^3^, Claus U. Pietrzik^1^

#### University Medical Center of the Johannes Gutenberg-University Mainz, Institute for Pathobiochemistry, Mainz, Germany, ^2^ University of Kentucky, Sanders-Brown Center on Aging Department of Pharmacology and Nutritional Sciences, Lexington, KY, USA, ^3^ University of Oslo (UiO) & Oslo University Hospital (OUS), Department of Neuro-Pathology, Oslo, Norway

##### **Correspondence:** Claus U. Pietrzik (pietrzik@uni-mainz.de)

*Fluids and Barriers of the CNS* 2017, **14(Supp 2)**:A52

According to the neurovascular hypothesis, impaired clearance of the amyloid-beta (Aβ) peptide across the blood–brain barrier (BBB) contributes to Alzheimer’s disease (AD) pathology. However, conflicting findings on the involvement of different Aβ transporters at the BBB and their expression in brain endothelium have questioned the role of LRP1 and Pgp at the BBB. As global knockout of Lrp1 in mice is lethal, there is a lack of appropriate models to study the function of LRP1. Moreover, the relevance of systemic Aβ clearance remains unclear as no BBB-specific knockout models had been available. We used in vitro and in vivo methods to quantify the rate of Aβ clearance across the BBB. With a novel Slco1c1-CreERT2 mouse, we generated the first brain endothelial-specific Lrp1 knockout mouse to accurately evaluate LRP1-mediated Aβ BBB-clearance in vivo. Using stereotactical injections of physiological concentrations of radiolabeled Aβ peptides, we were able to quantify the rate of LRP1-mediated clearance at the BBB in vivo. Crossing the LRP1 KO mice to the 5xFAD mouse model resulted in reduced plasma Aβ and elevated soluble brain Aβ leading to aggravated spatial learning and memory deficits, thus, emphasizing the importance of systemic Aβ elimination via the BBB. By combining primary mouse brain endothelial cells from these animals with Pgp inhibitors, we are able to identify the role of each transporter at the BBB for Aβ clearance in vitro. Dissecting the function of these transporters may provide new approaches for treatment and prevention of Aβ brain accumulation in AD.

Grant support: This work was supported by the EU Joint Programme—Neurodegenerative Disease Research (JPND) project (PROP-AD J.P. and C.U.P.) and the DFG (J.P. and C.U.P.)

## A53 Does perfluorooctanoic acid (PFOA) cross the blood–brain barrier?

### M. Surma^1^, Z. Giżejewski^2^, H. Zieliński^3^

#### ^1^Malopolska Centre of Food Monitoring, Faculty of Food Technology, University of Agriculture in Krakow, Krakow, Poland, ^2^Department of Gamete and Embryo Biology, Institute of Animal Reproduction and Food Research, Polish Academy of Sciences, Division of Reproductive Biology, Olsztyn, Poland, ^3^Department of Chemistry and Biodynamics of Food, Institute of Animal Reproduction and Food Research, Polish Academy of Sciences, Division of Food Science, Olsztyn, Poland

##### **Correspondence:** M. Surma (m.surma@ur.krakow.pl)

*Fluids and Barriers of the CNS* 2017, **14(Supp 2)**:A53

Perfluoroalkyl substances (PFASs) are classified as persistent and biaccumulative substances. They are metabolites of several polyfluorinated precursor compounds that are produced and used commercially. PFASs have been detected globally as pollutants in water, plants, foodstuffs, and in fish, birds, in mammals’ tissues as well as in human breast milk and blood. Moreover, they have been found in wildlife samples from all over the world thus suggesting their bio-accumulation in higher trophic levels in the food chains.

In this study, the possible PFASs bioaccumulation in tissues of animal origin was addressed with special focus on their ability to across the blood–brain barrier. Determination of selected perfluoroalkyl carboxilic acids (PFCAs) and perfluoroalkane sulfonates (PFSAs) concentration was performed in subcutaneous adipose and peritoneum tissues, internal organs (brain, liver) and tail tissue of free-living European beaver (Castor fiber L.) in Masurian Lakeland (NE Poland).

In a group of ten selected perfluoroalkyl substances only two perfluoroalkyl carboxilic acids (PFOA and PFNA) and one perfluoroalkane sulfonate (PFOS) were quantified. PFOA was detected in all analysed tissue samples in both female and male beavers in a range from 0.55 to 0.98 ng g^−1^ ww whereas PFOS was identified in all analyzed female beaver tissues and only in liver, subcutaneous adipose and peritoneum tissues of male beavers at the concentration level from 0.86 to 5.08 ng g^−1^ ww. PFNA was only identified in female beaver tissues (liver, subcutaneous adipose and peritoneum) in a range from 1.50 to 6.61 ng g^−1^ ww.

In this study for the first time, the accumulation of PFOA in brain tissue of beavers was shown. This finding may suggest the ability of PFOA to across the blood–brain barrier but the potential impact of this phenomenon in beavers is still unknown. In contrast, PFOS has limited potential to cross the blood–brain barrier since this compound was only detected in one beaver. We presume that the results of the present study, although of basic and preliminary character, demonstrate the bio-accumulation of PFOA, and to lesser extent PFOS and PFNA, in tissue samples collected from both female and male beavers living in area known as green lungs of Poland.

This research was performed within the EU project REFRESH (FP7-REGPOT-2010-1-264105).

## A54 Impact of leptin on choroid plexus response to systemic LPS-induced inflammation in ewes

### Aleksandra Szczepkowska^1^, Marta Kowalewska^1^, Agata Krawczynska^2^, Andrzej P. Herman^2^, Janina Skipor^1^

#### Institute of Animal Reproduction and Food Research, Polish Academy of Sciences, Olsztyn, Poland, ^2^ The Kielanowski Institute of Animal Physiology and Nutrition, Polish Academy of Sciences, Jablonna n/Warsaw, Poland

##### **Correspondence:** Aleksandra Szczepkowska (a.szczepkowska@pan.olsztyn.pl)

*Fluids and Barriers of the CNS* 2017, **14(Supp 2)**:A54

In sheep, the concentrations of leptin in blood plasma and the cerebrospinal fluid (CSF) are photoperiodically differentiated, increase in long days (LD) and decrease in short days (SD). Moreover, there is also photoperiodic differentiation in hypothalamic sensitivity to leptin that administered intracerebroventricularly suppresses appetite in SD but not LD. There is abundant expression of short isoform of leptin receptor (Ob-Ra) in the choroid plexus (CP) linked with leptin entry into CSF and lower expression of long isoform (Ob-Rb), a primary functional isoform. Moreover, most of the immune cells infiltrating CP during inflammation express Ob-Rb. Considering modulatory effect of leptin on inflammatory response and angiogenesis we hypothesized that leptin modulates CP response to immune stress and that this effect is photoperiodically differentiated.

Studies were performed on adult ewes kept in natural LD (n=24) and SD (n=24), treated with: saline (control), LPS (400 ng/kg), leptin (20 μg/kg) and LPS/leptin. Ewes were sacrificed 3 h after LPS/saline and 2.5 h after leptin/saline. Leptin receptors mRNA expression (*Ob-Ra, Ob-Rb*) and Ob-Rb activation pathway (*Jak2, Stat3, Socs3*), Toll-like receptors (*Tlr2, Tlr4*), cytokines and their receptors (*Il1B*, *Il1r1*, *Il1r2*, *Il1ra*, *Il6*, *Il6s*t*)*, chemokine (*Ccl2*) and factors linked with CSF secretion: aquaporin 1 *(Aqp1),* Claudin-2 (*Cldn2*) and system of vascular endothelial growth factor *(Vegf, VegfR1, VegfR2*) were analyzed by qPCR. Reactions on photoperiod and treatments were monitored by serum level of prolactin, cortisol and leptin.

Changes in leptin receptors were observed only during SD, when leptin did not affect but LPS reduced (p<0.05) mRNA expression of *Ob-Rb*. CP responded on LPS treatment, through mRNA elevation of all examined cytokines/cytokines receptors and chemokine, but for *Tlr2*, *Il1B, Il1R1, Il1Ra, Il6* and *Socs3* this reaction was significantly higher during SD vs. LD. Leptin increased (p<0.05) the response of CP to immune stress for *Il1r2*, *Il1ra*, *Il6*, *Il6st*, *Ccl2, Jak2 and Socs3,* during LD *and Il1B* during SD. LPS treatment decreased expression of *Cldn2* in SD and LD. Leptin did not affect *Aqp1* and *Cldn2*. In case of VEGF/VEGFR system, the impact of LPS and leptin was photoperiodically differentiated. LPS did not affect *Vegf120* and *Vegf164*, however increased (p<0.05) *VegfR1* and *VegfR2* but only in SD. In SD leptin increased (p<0.05) *Vegf164* and *VegfR1* and increasing tendency was observed for *Vegf120* (p= 0.06). During LD *VegfRs* increased only in leptin/LPS group. This results, confirmed hypothesis that leptin modulates CP response to immune stress in photoperiodically differentiated way.

Funded by the National Science Centre of Poland, grant SONATA no 2013/11/D/NZ9/02536. Participation of AS, MK and JS was supported by statutory research founds of Ministry of Higher Education.

## A55 Pericyte HIF-1 deficiency modulates stroke outcome: implications for a therapeutic time window of HIF-1 intervention

### Chih-Chieh Tsao^1,2^, Nicole Kachappilly^2^ & Omolara Ogunshola^1,2^

#### Zurich Center of Integrative Human Physiology, University of Zurich, Zurich, Switzerland, ^2^ Institute of Veterinary Physiology, University of Zurich, Zurich, Switzerland

##### **Correspondence:** Chih-Chieh Tsao (chih-chieh.tsao@uzh.ch)

*Fluids and Barriers of the CNS* 2017, **14(Supp 2)**:A55

Activation of hypoxia inducible factor-1 (HIF-1) is crucial for cells to adapt to hypoxic/ischemic stresses. For decades, HIF-1 stroke research has mainly been limited to neurons with stabilization of HIF-1 suggested as a potential neuroprotective strategy. However, successful employment of such strategy means the impact of HIF-1 induction on non-neuronal cells needs to be thoroughly understood during cerebral ischemia. Pericytes play a key regulatory and structural role in the ischemic blood–brain barrier (BBB). However, the consequences of altered pericyte HIF-1 signaling during stroke remain unknown.

Recently, we have generated a mouse line with pericyte-targeted HIF-1α knockout (KO) under the control of the inducible Cre/Lox system. The mouse line enables us to specifically explore effects of pericyte-mediated HIF-1 signaling on stroke outcome and BBB modulation after ischemic insult. We subjected pericyte HIF-1 KO and WT mice to transient middle cerebral artery occlusion (tMCAo) for 45 mins using intraluminal filament method followed by reperfusion up to 14 days. Ischemic damage, BBB permeability, neurological scores were assessed. Intriguingly, our data suggested stabilization of HIF-1 in pericytes differentially modulates barrier integrity and stroke outcome during stroke progression. Firstly, at 72h post-tMCAo HIF-1 deletion reduced IgG extravasation and Evans blue leakage. Although overall infarct volume was unaffected, reduced neuronal degeneration, less cell apoptosis and more astrocyte activation were observed in HIF-1 KO peri-infarct cortex by immunostaining. Therefore, HIF-1 deletion preserves barrier integrity and improves stroke outcome at the subacute stage. Surprisingly however, at 14 days reperfusion, reversal of positive effects of HIF-1 deletion was seen. HIF-1 KO mice exhibited higher blood vessel density compared to WT controls in correlation with increased permeability. Furthermore, enhanced brain shrinkage and Flurojade C-positive processes were observed in KO mice. Taken together, our results highlight a time-dependent effect of pericyte HIF-1 stabilization on BBB regulation after insult. The switch from maintenance of vascular homeostasis to negative outcome suggests a therapeutic time window should be considered when targeting HIF-1 for stroke therapy.

Grant support: Swiss National Science Foundation grant (31003A_150062) to Omolara Ogunshola

## A56 Mechanisms of CNS viral seeding by transm igration of HIV + CD14 + CD16 + monocytes across the blood brain barrier: establishment and reseeding of viral reservoirs contributing to HAND

### Mike Veenstra^1^, Rosiris Leon Rivera^1^, Dionna W. Williams^2,^ Susan Morgello^3^, Joan W. Berman^4^

#### ^1^Department of Pathology, Albert Einstein College of Medicine, Bronx, New York, NY, USA, ^2^Department of Molecular and Comparative Pathobiology, Johns Hopkins University School of Medicine, Baltimore, MD, USA, ^3^Departments of Neurology, Neuroscience, and Pathology, Icahn School of Medicine at Mount Sinai, New York, NY, USA, ^4^Departments of Pathology, and Microbiology and Immunology, Albert Einstein College of Medicine, Bronx, New York, NY, USA

##### **Correspondence:** Joan W. Berman (joan.berman@einstein.yu.edu)

*Fluids and Barriers of the CNS* 2017, **14(Supp 2)**:A56

HIV associated neurocognitive disorders (HAND) develop in >50% of HIV infected individuals, despite successful combined antiretroviral therapy (cART). HAND is mediated by mature CD14^+^CD16^+^ monocytes that transmigrate across the blood–brain barrier (BBB) in response to CCL2, a chemokine highly elevated in the CNS of HIV infected people, and establish viral reservoirs and an inflammatory and toxic environment in the CNS, leading to HIV neuropathogenesis. Our laboratory showed that transmigration of mature monocytes across the BBB is significantly greater in people with HAND than those without HAND, in part due to increased expression of CCR2, the CCL2 receptor. Additionally our laboratory showed the junctional proteins JAM-A and ALCAM are significantly increased on mature monocytes of HIV-infected people, and that these proteins are essential to transmigration across the BBB. We now show for the first time in HIV-infected individuals by FACS detection of cells that are infected with HIV, HIV^+^CD14^+^CD16^+^ monocytes, and cells that are not infected but that have been exposed to viral proteins and inflammatory mediators, HIV^exp^CD14^+^CD16^+^ monocytes. We demonstrate that HIV^+^CD14^+^CD16^+^ monocytes express higher JAM-A and ALCAM than HIV^exp^CD14^+^CD16^+^ monocytes. Additionally, we demonstrate that primary human HIV^+^CD14^+^CD16^+^ monocytes preferentially transmigrate across the BBB in response to CCL2 in comparison to HIV^exp^CD14^+^CD16^+^ monocytes. We propose that this is mediated, in part, due to increased JAM-A, ALCAM, and CCR2 on the HIV^+^ mature monocytes. Antibodies against JAM-A and ALCAM, and the novel CCR2/CCR5 dual inhibitor cenicriviroc, prevented or significantly reduced preferential transmigration of HIV^+^CD14^+^CD16^+^ monocytes. This indicates that JAM-A, ALCAM, and CCR2 may be potential therapeutic targets to block entry of these infected cells into the brain, and prevent or reduce the establishment and replenishment of viral reservoirs within the CNS, resulting in decreased neuroinflammation, toxicity, and HAND.

This work was supported by U.S. National Institutes of Health Grants R01MH075679 (J.W.B.), R21MH102113-01A1 (to J.W.B), R01MH090958 (J.W.B.), 1R01MH112391 (J.W.B), U24MH100931 (Manhattan HIV Brain Bank), P30AI124414 (Einstein-Rockefeller-CUNY Center for AIDS Research), TL1TR001072 (Einstein-Montefiore CTSA), and by the eCLIPSE program fellowship supported by the Burroughs Wellcome Foundation Grant program “Unifying Population and Laboratory Science”(MV).

## A57 Crosstalk between brain and periphery by small extracellular vesicles

### Ursula Wyneken and Luis Federico Batiz

#### Centro de Investigación Biomédica (CIB), Facultad de Medicina, Universidad de los Andes, Santiago, Chile

##### **Correspondence:** Ursula Wyneken (uwyneken@uandes.cl)

*Fluids and Barriers of the CNS* 2017, **14(Supp 2)**:A57

Small extracellular vesicles (EVs), including microvesicles (originated from the plasma membrane) and exosomes (originated in the endocytic pathway), have emerged as central players in intercellular communication. They are released into the extracellular space by almost every cell type and can functionally regulate target cells due to their bioactive cargo (i.e. proteins, lipids, nucleic acids and carbohydrates), which varies under different physiological or pathological conditions. However, the EV-mediated bi-directional communication between the CNS and the vascular system remains enigmatic, as well as the regulation of this process under chronic stress. We have focused on astrocyte-derived EVs and their interaction with and effects on immature neurons, neural precursors, and their possible transfer to the blood. Besides typical EV markers (such as CD63, TSG101, flotillin), astrocyte-derived EVs contain microRNA 26a (miR26a) and aldolase C, a glycolytic enzyme expressed selectively in forebrain astrocytes. We have used *in utero* electroporation of rat brains to transfer aldolase C-GFP to astrocytes. Interestingly, the tagged enzyme is detected not only in CSF-derived EVs but also in blood serum-derived EVs. Moreover, the protein cargo of serum EVs, including aldolase C and Glial Fibrillary Protein (GFAP), varied under selective stress conditions. The EV-mediated transfer of aldolase C-GFP and of miR26a to immature neurons had morphological consequences that were mediated by miR26a while its transfer to neural precursor cells and endothelial cells is under study. Thus, *in vivo* and *in vitro* studies show that brain-derived EVs can be transferred through brain barriers into the blood. Conversely, blood-borne EVs can reach the neural tissue thus acting as major regulators of brain function. The potential EV-mediated transfer of signals from the periphery to adult neurogenic niches could be explored in the future with therapeutic purposes.

**Acknowledgements:** Fondecyt 1140108 (UW) and 1141015 (LFB)

## A58 Blood–brain barrier dynamics of nanotechnological drug carriers in animal model of temporal lobe epilepsy

### Canan Uğur Yılmaz^1^, Nurcan Orhan^2^, Arzu Temizyürek^3^, Müge Atış^4^, Uğur Akcan^5^, Rouhollah Khodadust^6^, Nadir Arıcan^7^, Mutlu Küçük^1^, Candan Gürses^8^, Bülent Ahıshalı^9^, Serkan Emik^10^, Mehmet Kaya^11^

#### ^1^Department of Laboratory Animals Science, Aziz Sancar Institute of Experimental Medicine, Istanbul University, Istanbul, Turkey, ^2^Department of Neuroscience, Aziz Sancar Institute of Experimental Medicine, Istanbul University, Istanbul, Turkey, ^3^Center for Life and Sciences and Technologies, Bosphorus University, Istanbul, Turkey., ^4^Department of Cellular and Molecular Medicine, Medical Faculty, Koc University, Istanbul, Turkey, ^5^Department of Neuroscience, Medical Faculty, Koc University, Istanbul, Turkey, ^6^Department of Biotechnology, Medical Faculty, Koc University, Istanbul, Turkey, ^7^Department of Forensic Science, Istanbul Faculty of Medicine, Istanbul University, Istanbul, Turkey, ^8^Department of Neurology, Istanbul Faculty of Medicine, Istanbul University, Istanbul, Turkey, ^9^Department of Histology and Embryology, Istanbul Faculty of Medicine, Istanbul University, Istanbul, Turkey, ^10^Department of Chemical Technologies, Faculty of Engineering, Istanbul University, Istanbul, Turkey, ^11^Department of Physiology, Medical Faculty, Koc University, Istanbul, Turkey

##### **Correspondence:** Mehmet Kaya (mkaya942@gmail.com)

*Fluids and Barriers of the CNS* 2017, **14(Supp 2)**:A58

Although there are many antiepileptic drugs currently used for the treatment of epilepsy, drug resistance is still a main problem with the underlying mechanisms still poorly understood. One of the major reasons for the failure of antiepileptic drugs in resistant epilepsy is the blood–brain barrier (BBB). In this study, temporal lobe epilepsy (TLE) was induced in adult Wistar rats using kainic acid. Glucose coated gold nanoparticles (GNP) were loaded with lacosamide (LCD) and labeled with a fluorescent probe and presented into the circulation to be transported into the brain for treatment. The amount of GNP in the brain tissue was measured with inductively coupled plasma mass spectrometry, and fluorescent signals were observed by in vivo imaging. Electroencephalography (EEG) was recorded from the hippocampus of animals. Electron microscopic evaluation was performed to show GNP in the brain parenchyma. The main amplitude of the EEG records returned to the basal values right after GNP-LCD administration in animals with TLE. A time-related increase in the fluorescent signals was observed by GNP-LCD injection in the brains of animals with TLE. When the amount of GNP either loaded or not loaded with LCD was measured in the brain parenchyma of animals with or without TLE, the amount of GNP not loaded with LCD was found to be significantly higher in animals with TLE (p<0.01). Ultrastructurally, GNP and GNP-LCD transported across BBB were observed to be distributed in a diffuse pattern in the brain parenchyma of animals with or without TLE and had a tendency to aggregate in clumps. The results of this study show that GNP, a nanocarrier, may play an effective role in transferring effective doses of LCD into the brain in TLE therapy and hence, may represent a new approach in the treatment of epilepsy.

**Keywords:** Gold nanoparticle, Drug delivery, Blood–brain barrier, Lacosamide, Temporal lobe epilepsy

## A59 Altered expression of miRNAs may be instrumental in triggering the blood brain barrier disruption in hyperammonemia related or not related to hepatic failure

### Magdalena Zielińska, Marta Obara-Michlewska, Krzysztof Milewski, Edyta Skonieczna, Inez Fręśko

#### Neurotoxicology Department, Mossakowski Medical Research Center, Polish Academy of Sciences, Warsaw, Poland

##### **Correspondence:** Magdalena Zielińska (mzielinska@imdik.pan.pl)

*Fluids and Barriers of the CNS* 2017, **14(Supp 2)**:A59

The pathophysiological mechanisms underpinning the blood brain barrier (BBB) dysfunction in hepatic encephalopathy (HE) are complex and remain to be fully unraveled. While the central role of hyperammonemia in the BBB impairment in HE remains undisputed, differences between the effects of ammonia and toxic events related to liver failure, including the inflammatory responses, are currently a matter of intensive investigations. Recent data indicate that functioning of BBB is modulated by the non-coding single-stranded RNAs-microRNAs (miRNAs). In this study the expression of miRNAs was profiled in cerebral cortices of rats subjected to simple hyperammonemia (HA) and hepatic failure in the thioacetamide (TAA) model using miRNA microarray technique. The ultimate goal was to analyze the roles of selected miRNAs in the development of BBB disruption by identifying their specific molecular targets. Altered expression of miRNAs was observed in the cortex of both TAA and HA rats. Target analysis revealed that several of the pro-inflammatory and related genes are targets of the downregulated miRNAs. The results suggest that the inflammation-related aspect of BBB damage in HE may be epigenetically regulated by miRNA in hyperammonemic conditions related or not related to liver dysfunction. Detailed analysis of the differences between miRNA responses in HA and TAA rats is under way and will be presented.

Grant support: National Science Centre grant 2015/19/B/NZ4/01902

## A60 Three areas where studies of the blood–brain barrier change patient care

### Edward A. Neuwelt

#### Oregon Health & Science University, Portland, OR 97239, USA

##### **Correspondence:** Edward A. Neuwelt (neuwelte@ohsu.edu)

*Fluids and Barriers of the CNS* 2017, **14(Supp 2)**:A60

Blood–brain barrier (BBB) research by our group has had a positive impact on improving survival and long-term outcomes in patients with primary and metastatic brain tumors in three areas: (1) brain tumor therapy, (2) neuroimaging, and (3) the prevention of platinum-induced ototoxicity.

1. BBB disruption (BBBD) chemotherapy. In a multi-institutional PCNSL clinical trial we showed that methotrexate-based chemotherapy in conjunction with BBBD results in successful and durable tumor control and outcomes that are comparable or superior to other PCNSL treatment regimens [1]. Prospective evaluation of PCNSL survivors who were treated with BBBD chemotherapy showed stable or improved cognitive status at a median follow-up of 12 years after diagnosis [2]. Patients receiving BBBD chemotherapy in our series showed equivalent cognition and neuroimaging outcomes compared to patients treated with high dose methotrexate without BBBD [3]. Current studies are evaluating immunotherapy with monoclonal antibodies (mAb) targeting B-lymphoma antigens as a mechanism to further improve the complete response rate and overall survival.
2. Novel magnetic resonance imaging (MRI) techniques for measuring changes in brain tumor vasculature and inflammation. In preclinical brain tumor studies, ferumoxytol improves the consistency of relative cerebral blood volume (rCBV) measurements in rats before and after treatment with anti-angiogenic and therapeutic mAbs [4]. At later time points, >24 h, ferumoxytol crosses the BBB in intracerebral lesions where it is taken up by activated microglia, astrocytes, and tumor-associated macrophages [5]. Neuroimaging with ferumoxytol provides a mechanism to differentiate tumor progression from pseudoprogression, which can resolve the clinical dilemma of continuation of effective therapy or transferring non-responders to novel therapies.
3. Chemoprotection and chemo-enhancement for brain tumor therapy. Platinum-based chemotherapy is used to treat a variety of childhood malignancies, but is associated with progressive and irreversible toxicities, in particular ototoxicity [6]. Research from our lab has shown that the thiols *N*-acetylcysteine (NAC) and sodium thiosulfate (STS) can protect against cisplatin-induced ototoxicity, and NAC is also protective against bone marrow and kidney toxicity. In patients with brain tumors treated with carboplatin based chemotherapy in conjunction with BBBD, we have shown that STS protects against carboplatin-based hearing loss and may also protect against carboplatin-induced severe thrombocytopenia [7]. To date, STS has been shown to be protective against cisplatin-induced hearing loss in children, and has not shown any evidence of tumor protection in children with localized disease.

1. Angelov L, Doolittle ND, Kraemer DF, Siegal T, Barnett GH, Peereboom DM, Stevens G, McGregor J, Jahnke K, Lacy CA, Hedrick NA, Shalom E, Ference S, Bell S, Sorenson L, Tyson RM, Haluska M, Neuwelt EA. Blood–brain barrier disruption and intra-arterial methotrexate-based therapy for newly diagnosed primary CNS lymphoma: a multi-institutional experience. J Clin Oncol. 2009;27:3503–9.
2. Doolittle ND, Dósa E, Fu R, Muldoon LL, Maron LM, Lubow MA, Tyson RM, Lacy CA, Kraemer DF, Butler RW, Neuwelt EA. Preservation of cognitive function in primary CNS lymphoma survivors a median of 12 years after enhanced chemotherapy delivery. J Clin Oncol. 2013;31:4026–7.
3. Doolittle ND, Korfel A, Lubow MA, Schorb E, Schlegel U, Rogowski S, Fu R, Dósa E, Illerhaus G, Kraemer DF, Muldoon LL, Calabrese P, Hedrick N, Tyson RM, Jahnke K, Maron LM, Butler RW, Neuwelt EA. Long-term cognitive function, neuroimaging, and quality of life in primary CNS lymphoma. Neurology. 2013;81:84–92.
4. Gahramanov S, Muldoon LL, Li X, Neuwelt EA. Improved perfusion MR imaging assessment of intracerebral tumor blood volume and antiangiogenic therapy efficacy in a rat model with ferumoxytol. Radiology. 2011;261:796–804.
5. McConnell HL, Schwartz DL, Richardson BE, Woltjer RL, Muldoon LL, Neuwelt EA. Ferumoxytol nanoparticle uptake in brain during acute neuroinflammation is cell-specific. Nanomedicine. 2016;12:1535–42.
6. Neuwelt EA, Brock P. Critical need for international consensus on ototoxicity assessment criteria. J Clin Oncol. 2010;28:1630–2.
7. Doolittle ND, Muldoon LL, Culp AY, Neuwelt EA. Delivery of chemotherapeutics across the blood–brain barrier: challenges and advances. Adv Pharmacol. 2014;71:203–43.

## A61 The effect of lidocaine on the surface charge of brain endothelial cells

### Ana Raquel Santa Maria, Ana Rita Bras, Dóra Lipka, Sándor Valkai, András Kincses, András Dér, Maria A. Deli

#### Institute of Biophysics, Biological Research Centre, Hungarian Academy of Sciences, Szeged 6726, Hungary

##### **Correspondence:** Ana Raquel Santa Maria (anaraquel.santamaria@brc.mta.hu)

*Fluids and Barriers of the CNS* 2017, **14(Supp 2)**:A61

Plasma membrane surface charge originating from negatively charged lipid head groups and the glycocalyx play an important role as a defence system at the blood–brain barrier. It can regulate permeability across brain endothelial cells including drug transport. Lidocaine, a cationic and lipophilic molecule used as anaesthetic and antiarrhythmic drug can change the surface charge of lipid membranes, and this is one of the hypothesized ways of its mode of action. However, the direct action of lidocaine on surface charge of endothelial cells was not yet measured. Our aim was to study the direct effect of lidocaine on the surface charge and barrier properties of brain endothelial cells.

Surface charge of hCMEC/D3 human brain endothelial cells was measured by Zetasizer Nano (Malvern). Barrier properties of hCMEC/D3 monolayers were evaluated by measuring transendothelial electrical resistance, permeability for hydrophilic marker molecules, and morphology of intercellular junctions stained by immunochemistry.

Lidocaine changed the surface charge of the brain endothelial cells, the negative zeta potential became more positive. After both short term (30 min) and 2-day lidocaine treatment a decrease in the electrical resistance of brain endothelial monolayers was found indicating an increased ion movement across the paracellular space, but no significant change was detected in the permeability for large paracellular marker molecules or in the morphology of interendothelial junctions. We suggest that the effect of lidocaine is due to a change in the lipid part of the plasma membrane, which became more positive after lidocaine treatment. As a summary, we could demonstrate by direct measurements, that lidocaine makes the surface charge of human brain endothelial cells more positive. While lidocaine may not damage directly the blood–brain barrier in vivo, a significant surface change in brain endothelial cells may modulate brain penetration of charged drug molecules.

Grant support: European Training Network Brain Barriers Training H2020-MSCA-ITN-2015 675619, GINOP-2.3.2-15-2016-00001, GINOP-2.3.2-15-2016-00060

